# Pathogenesis of *Staphylococcus haemolyticus* on primary human skin fibroblast cells

**DOI:** 10.1080/21505594.2020.1809962

**Published:** 2020-08-30

**Authors:** Hala O. Eltwisy, Medhat Abdel-Fattah, Amani M. Elsisi, Mahmoud M. Omar, Ahmed Aly Abdelmoteleb, Mohamed A. El-Mokhtar

**Affiliations:** a Department of Microbiology, Faculty of Science, Beni-Suef University, Beni-Suef, Egypt; b Department of Microbiology and Botany, Faculty of Science, Beni-Suef University, Beni-Suef, Egypt; c Department of Pharmaceutics and Industrial Pharmacy, Beni-Suef University, Beni-Suef, Egypt; d Department of Pharmaceutics and Industrial Pharmacy, Deraya University, El-Minia, Egypt; e Department of General Surgery, Faculty of Medicine, Assiut University, Assiut, Egypt; f Department of Medical Microbiology and Immunology, Faculty of Medicine, Assiut University, Assiut, Egypt

**Keywords:** *Staphylococcus haemolyticus*, diabetic foot ulcer, primary human skin fibroblast cells, bacterial invasion, pathogenesis

## Abstract

**Staphylococcus haemolyticus:**

(*S. haemolyticus*) is one of the Coagulase-negative staphylococci (CoNS) that inhabits the skin as a commensal. It is increasingly implicated in opportunistic infections, including diabetic foot ulcer (DFU) infections. In contrast to the abundance of information available for *S. aureus* and *S. epidermidis*, little is known about the pathogenicity of *S. haemolyticus*, despite the increased prevalence of this pathogen in hospitalized patients. We described, for the first time, the pathogenesis of different clinical isolates of *S. haemolyticus* isolated from DFU on primary human skin fibroblast (PHSF) cells. Virulence-related genes were investigated, adhesion and invasion assays were carried out using Giemsa stain, transmission electron microscopy (TEM), MTT and flowcytometry assays. Our results showed that most *S. haemolyticus* carried different sets of virulence-related genes. *S. haemolyticus* adhered to the PHSF cells to variable degrees. TEM showed that the bacteria were engulfed in a zipper-like mechanism into a vacuole inside the cell. Bacterial internalization was confirmed using flowcytometry and achieved high intracellular levels. PHSF cells infected with *S.haemolyticus* suffered from amarked decrease in viability and increased apoptosis when treated with whole bacterial suspensions or cell-free supernatants but not with heat-treated cells. After co-culture with PBMCs, *S. haemolyticus* induced high levels of pro-inflammatory cytokines. This study highlights the significant development of *S. haemolyticus*, which was previously considered a contaminant when detected in cultures of clinical samples. Their high ability to adhere, invade and kill the PHSF cells illustrate the severe damage associated with DFU infections.

**Abbreviations:**

CoNS, coagulase-negative staphylococci; DFU, diabetic foot ulcer; DM, diabetes mellitus; DMEM, Dulbecco’s Modified Eagle Medium; MTT, 3-(4, 5-dimethylthiazolyl-2)-2, 5-diphenyltetrazolium bromide; PBMCs,peripheral blood mononuclear cells; PHSF, primary human skin fibroblast; CFU, colony-forming unit.

## Introduction

*Staphylococcus haemolyticus* (*S. haemolyticus*)is one of the coagulase-negative staphylococci (CoNS) that inhabit the skin as a commensal. It is increasingly implicated in opportunistic infections in immunocompromised patients, particularly in hospitalized patients and those with medical implants worldwide [1]. It is the second most frequent CoNS isolated from infected clinical samples,particularly blood cultures of patients with sepsis following *S. epidermidis* [2].

*S. haemolyticus* causes severe infections in several body systems including meningitis, endocarditis, prosthetic joint infections and bacteremia and is prevalent in the hospital environment and on the hands of healthcare workers. *S. haemolyticus* is also known to cause septicemia, peritonitis, otitis media and diabetic foot ulcer (DFU) infections [3,4].

Diabetes mellitus (DM) is a metabolic disease that is associated with increased susceptibility to bacterial infections. Patients usually suffer from infected foot ulcers, which increases the complexity of their treatment. About 15% of patients with DM develop foot ulcers that may progress to osteomyelitis and amputation [5]. These bacterial infections are usually caused by the coagulase-positive *Staphylococcus aureus* (*S. aureus*) and also the emerging CoNS, including *Staphylococcus epidermidis* (*S. epidermidis*) and *S. haemolyticus* [6].

A characteristic feature of *S. haemolyticus* is its ability to form biofilms, which play an essential role in the establishment of infections. The produced exopolysaccharides can inhibit the growth of other bacteria and also decrease their ability to form biofilms [7]. This specieshas gained an increased clinical significance due to its genome plasticity, which allowed a great adaptation and development of resistance to different antibiotics, including methicillin and its ability to survive in the hospital environment [8]. The remarkable ability of *S. haemolyticus* to acquire antibiotic resistance, especially to oxacillin, limits the available therapeutic options for catheter-related infections caused by methicillin resistant *S. haemolyticus* isolates and may predispose to sepsis and increase patient’s morbidity and mortality [9,10]. *S. haemolyticus* and *S. aureus* have >99.9% identities in the sequences of *beta-lactamase* and *qacA* genes, pointing to the possibility of interspaces exchange of the genetic elements responsible for resistance to antibiotics [11].

In contrast to the abundance of information available for *S.aureus* and *S. epidermidis*, little is known about the pathogenicity and virulence factors of *S.haemolyticus*, despite the increased prevalence of this pathogen in immunocompromised patients [12].

The aim of this study was to describe, for the first time, the pathogenesis of clinical isolates of *S. haemolyticus*, isolated from DFU on PHSF cells. Virulence-related genes were investigated, adhesion and invasion assays were carried out usingGiemsa stain, transmission electron microscopy (TEM), MTT and flow cytometry assays. The potential cytotoxic and apoptotic effects induced by *S. haemolyticus* were investigated. Also, changes in cytokine profile in response to infection with *S. haemolyticus* were shown. Our results contribute to a better understanding of the pathomechanisms of *S. haemolyticus* infections and fill a gap in the literature regarding the CoNS. Based on our observations, microbiology laboratories should consider *S. haemolyticus* a critical opportunistic pathogen and infections caused by this organism, particularly in critically ill patients, should be seriously managed.

## Materials and methods

### Ethics statement

The study protocol was approved by the Ethics Committee of the Faculty of Medicine, Assiut University (Assuit, Egypt) and conducted in accordance with the provisions of the Declaration of Helsinki (approval number 17-300-379). Informed written consent was obtained from study participants.

### Skin punch biopsy explant culture for preparation of the PHSF cells

Isolation of PHSF cells from skin punch biopsy was performed as described before, with minor modifications [13,14]. Skin biopsy specimens were obtained from anonymous healthy donors (aged between 20 and 35 years) during surgery procedures of abdominal dermolipectomy. Skin fragments were immersed in 70% ethanol and washed 3 times by phosphate buffer saline (PBS). Fibroblast harvesting was done by explant technique. The dermis and epidermis were isolated from the subcutaneous tissue and fragmented into 5 mm^2^ pieces with scalpels and scissors. These fragments (epidermis upward and dermis downward) were laid onto the surface of a 6-well plate, which was pre-coated with 0.2% gelatin solution (Sigma, Germany). We used a coverslip to hold down the skin pieces that helped the adhesion of fragment to the gelatin-coated plates. DMEM supplemented with 30% (v/v) fetal bovine serum (FBS) and 1% (v/v) penicillin-streptomycin (all from Gibco, USA) were added to each well and medium was changed every 2–3 days. After 20 days, fibroblasts were sub-cultured using trypsin-EDTA (0.25%) (Gibco, USA).

### Bacterial isolates and species identification

From March 2018 through February 2019, a prospective study of patients with diabetic foot ulcers admitted to the diabetic foot unit at Assiut University Hospital was conducted. One hundred patients were enrolled. After wound debridement, the wound base was swabbed using a cotton swab moistened with sterile 0.9% NaCl solution for bacterial culture, and samples were sent immediately to the microbiology department. Swabs were enriched into brain heart infusion broth (Becton Dickinson GmbH, Heidelberg, Germany) then streaked onto enriched and selective bacterial culture plates.

Staphylococci were identified using the VITEK2 Microbiology automated system (VITEK®2, BioMerieux). Moreover, the identity of staphylococciwas confirmed using multiplex PCR as described previously [15]. During the study period, ten *S. haemolyticus* isolates were obtained from the cultures of DFUs from ten different infected patients. In these infected ulcers, only *S. haemolyticus* was detected. We referred to these isolates as SH1 to SH10 in our infection experiments.In some experiments, four different*S. aureus*clinical isolates were used as controls and for comparison purposes (referred to as SA12, SA13, SA14 and SA22). These isolates were also recovered from patients suffering from severe deep DFU infections from the same hospital. Patients’ demographic characteristics and the antimicrobial resistance profile of the isolated *S. haemolyticus* used in the studyare shown in supplemental table S1. In addition, *S. haemolyticus* (ATCC 29,970) was used as a control in the phenotypic functional experiements (referred to in most experiments as control *S. haemolyticus*).

Methicillin resistance was detected by initial phenotypic screening for reduced susceptibility of the isolates to cefoxitin (30 µg) and oxacillin (1 μg) disks (Mast, UK) by the disk diffusion method, according to Clinical and Laboratory Standards Institute (CLSI) guidelines. Then, methicillin resistance was confirmed by amplification of the *mecA* gene by PCR assay using specific primers proposed by Pinheiro, Brito [15].

### Hemolysis assay

The hemolytic activity of *S. haemolyticus* isolates was analyzed according to the protocol of Cremet, Broquet [16],using a clinical isolate of *S. aureus* as a control. Hemolytic activity was measured spectrophotometrically by using a microplate reader (Epoch™ Microplate Spectrophotometer, BioTek, USA). Briefly, the hemolytic activity of the strains was quantified in human blood diluted to 10% (v/v) in PBS. For these experiments, 200 μl of overnight bacterial suspension with a concentration of about 10^8^ CFU/ml in PBS was added to 200 μl of the erythrocyte suspension and the mixture was incubated for 5 h. Then samples were centrifuged at 1500 xg for 5 min. Hemoglobin release in the supernatant was observed by measuring absorbance at 450 nm. Blood treated with 1% Triton X-100 was used as a positive control (100% hemolysis) and blood treated with PBS was used as a negative control (0% hemolysis).

### Detection ofenterotoxin, hemolysin, fibronectin-binding protein and exfoliativegenes

PCR was used for the detection of different virulence-associated genes; *sea, seb, sec, sed, see, seg, seh, α-hemolysin, fnbA, fnbB, eta* and *etb*. Target genes and primer sequences are detailed in Table 1.

**Table 1.** List of primers used for the detection ofthe enterotoxins, hemolysin, fibronectin-binding protein, and exfoliative toxins genes.

Group name	Primer name	Gene name	Sequence	Size (bp)	References
*Enterotoxins*	*sea-1* *sea-2*	*Enterotoxin A*	**F:**TTGGAAACGGTTAAAACGAA **R:**GAACCTTCCCATCAAAAACA	120	[60]
	*seb-1* *seb-2*	*Enterotoxin B*	**F:**TCGCATCAAACTGACAAACG **R:**GCAGGTACTCTATAAGTGCC	478	
	*sec-1* *sec-2*	*Enterotoxin C*	**F:**GACATAAAAGCTAGGAATTT **R:**AAATCGGATTAACATTATCC	257	
	*sed-1* *sed-2*	*Enterotoxin D*	**F:**CTAGTTTGGTAATATCTCCT **R:**TAATGCTATATCTTATAGGG	317	
	*see-1* *see-2*	*Enterotoxin E*	**F:**CAAAGAAATGCTTTAAGCAATCTTAGGCCAC **R:**CTTACCGCCAAAGCTG	482	
	*seg-1* *seg-2*	*Enterotoxin G*	**F:**AATTATGTGAATGCTCAACCCGATC **R:**AAACTTATATGGAACAAAAGGTACTAGTTC	642	
	*seh-1* *seh-2*	*Enterotoxin H*	**F:**CAATCACATCATATGCGAAAGCAG **R:**CATCTACCCAAACATTAGCACC	376	
*Hemolysin*	*hla_haem-1* *hla_haem-2*	*α-hemolysin*	**F:**TGGGCCATAAACTTCAATCGC **R:**ACGCCACCTACATGCAGATTT	72	
*Fibronectin-binding protein*	*fnbA*	*Fibronectin-Binding Proteins A*	**F:**CCCTCTTCGTTATTCAGCC **R:**CAGGAGGCAAGTCACCTTG	422	[73]
	*fnbB*	*Fibronectin-Binding Proteins B*	**F:**TAAATCAGAGCCGCCAGTGGAG **R:**GTCCTTGCGCTTGACCATGTTC	416	[74]
*Exfoliative toxins*	*eta F* *eta R*	exfoliative toxins A	**F:**ACTGTAGGAGCTAGTGCATTTGT **R:**TGGATACTTTTGTCTATCTTTTTCATCAAC	190	[75]
	*etb F* *etb R*	exfoliative toxins B	**F:**CAGATAAAGAGCTTTATACACACATTAC **R:**AGTGAACTTATCTTTCTATTGAAAAACACTC	612	

### Preparation of bacteria for infection experiments

In our infection experiments, fresh bacterial suspensions were prepared according to the following protocol. Twenty-four hours prior to experiments, 2–3 colonies of *S. haemolyticus* were transferred from blood agar into LB broth and incubated at 37°C. The overnight bacterial culture was centrifuged at 1500 xg for 5 min, the supernatant was removed and the cell pellet was suspended in 2 ml PBS. For preparation of bacterial supernatant, the supernatant of bacteria was filtered through 0.2 µm filter. For infection assays,PHSF cells were plated at a density of 10^5^ cells/wells in 12-well tissue culture plates with 1 ml of DMEM cell culture medium and 1% FBS without antibiotics (invasion medium). Before infection, cells were washed with PBS and theinvasion medium was added and kept for 1 h at 37°C. In all infection experiments, the optical density of bacteria was adjusted at OD_600_ = 0.2, which is equivalent to a multiplicity of infections (MOIs) of 10 bacteria per cell.

### Adhesion assay using Giemsa stain

Adhesion of *S. haemolyticus* to PHSF cells was tested. To do that, washed bacterial suspension was added to cells on coverslips at MOI of 10, and cells were incubated for 3 h at 37°C. Coverslips were washed 6 times to remove non-adhering bacteria by immersing the coverslips in saline. 500 μl of Giemsastain (10%) was added to each well for 30–40 min at room temperature. Coverslips were washed to remove the excess of the stain or stain debris. Cells were fixed by adding 200 μl of 70% methanol for 5 min at −20°C. Then, coverslips were washed by PBS to remove methanol and left to air dry. Coverslips were examined microscopically (magnification of x100) and the number of adherent bacteriawascountedin 20 randomly selected microscopic fields and averaged. Very strong adhesion (++++) represented> 2500 adherent bacterial cells; strong adhesion (+++) represented 2500–1000 adherent bacteria; moderate adhesion (++) represented 1000 − 100 adherent bacteria and weak adhesion (+) represented less than 100 adherent bacteria,similar to the method reported by Guglielmetti, Taverniti [17].

### Transmission electron microscopy

Electron microscopy was used to visualize the adhesion and invasion of *S. haemolyticus* into the PHSF cells. Cells were grown in T75 Flask and infected with *S. haemolyticus* as described above. After 15, 60 and 90 min post-infection, cells were washed three times with PBS and harvested by trypsinization. They were centrifuged at 1200 xg for 5 min, fixed with 2% glutaraldehyde, washed two times with PBS and postfixed with 1% OsO_4_ for 2 h. Cells were embedded in 2% agar for 15 min. Sample was dehydrated with sequential ethanol baths (30 to 100%) for 10 min, and embedded in Epon 812 resin with a 48 h polymerization time at 70°C. By using an ultramicrotome, the embedded samples were sliced. Imageswere performed with a JEM 100 CX11 transmission electron microscope [18,19].

### Flow cytometry invasion assay

The ability of *S. haemolyticus* to invade the PHSF cells was also analyzed by flow cytometry invasion assay as described elsewhere [20], with minor modifications. Suspensions of FITC-labeled bacteria(OD_600_ = 0.2) were added to PHSF cells for 1 h at 4°C to allow for sedimentation of bacteria and then changed to 37°C for 3 h, to allow for cellular invasion. Finally, PHSF cells were harvested, and 100 μg/ml lysostaphin (Sigma-Aldrich, Germany) in PBS was added for 25 min at ambient temperature to remove extracellular bacteria. PHSF cells were transferred to 5 ml round-bottom polystyrene FACS tubes (Falcon; BD, Heidelberg, Germany), pelleted, and resuspended in PBS. Cells were analyzed by flow cytometry (BD FACSCalibur, USA) to determine the frequency of FITC-labeled cells.

### Lysostaphin protection assay

To determine the colonization of PHSF cells by *S. haemolyticus*, we performed alysostaphin protection assay [21]. PHSF cells were infected with *S. haemolyticus*and incubated for 3 h in invasion medium. After three washes with PBS, lysostaphin(100 μg/ml) was added for 25 min. Cells were lysed by the addition of 1 ml 0.5% Triton X-100 (Sigma-Aldrich, Germany) in PBS for 20 min at 37°C. Tenfold serial dilutions of cell lysates in sterile H_2_O were plated onto Nutrient Agar (Oxoid) and incubated overnight at 37°C to quantify the intracellular bacteria by counting the colony-forming units (CFUs).

### *Effect of* S. haemolyticus *on PHSF cell proliferation rate*

The effect of *S. haemolyticus* on the proliferation rate ofPHSF cells was initially tested by using the MTT assay, as described by Saliba, Filloux [22]. Cells were seeded into 96-well plates at a density of 1 × 10^4^ cells/well. After 24 h, cells were treated with washed bacterial suspension, cell-free bacterial supernatant, or heated bacterial suspension. Heat killed bacteria were prepared by heating washed bacterial suspension at 65°C for 30 min using the Heat block (Thermo-mixer, Germany). Control wells contained 100 μl DMEM, 50 μl sterile broth instead of the added bacterial culture. Plates were incubated at 37°C, and cell viability was tested at different time points at 6, 18 and 24 h post-treatment. Cells were treated with 10 μl of 2 mg/ml MTT reagent (SERVA, Germany) in PBS for 4 h at 37°C. 100 μl of DMSO (Sigma-Aldrich, Germany) was added per well to dissolve the formazan crystals formed in the viable metabolically active cells. The absorbance of the resultant solution was measured at 570 nm using a microplate Spectrophotometer (EPOCH, USA). The percentage of cytotoxicity was calculated using the following formula:

$\%Cytotoxicity=\left(100-\frac{OD570\;of\;inflected\;culture}{OD570\;of\;control}\right)\times \;100$

### Detection of apoptosis by flow cytometry

To analyze the ability of *S. haemolyticus* to induce apoptosis in the PHSF cells, adherent PHSF cells were infected with washed *S. haemolyticus*. After 24 h, cells were de-attached by trypsin and apoptosis was detected by flow cytometry using Annexin V/PI staining (ApoFlowEx FITC Kit, exbio). Early apoptotic cells were stained with annexin V alone, whereas necrotic cells and late apoptotic cells stained with both annexin V and propidium iodide.

### *Effect of* S. haemolyticus *on cytokine production*

Peripheral blood mononuclear cells (PBMCs) were separated from heparinized whole venous blood by density gradient centrifugation using Histopaque®-1077 (Sigma-Aldrich, Germany) following manufacturer’s instructions. PBMCs (1 x 10^7^ cells/ml) were suspended inRPMI 1640 medium (Gibco, USA) and infected with washed bacterial suspensionof *S. haemolyticus* (MOI = 10) in 6 well plates at 37°C. PBMCs suspended in RPMI 1640 medium (Gibco, USA) without antibiotics served as a control to establish basal cytokine levels. After 6 and 24 h of incubation, cells were washed 2 times with PBS and incubated with lysostaphinthen subjected to RNA extraction (GeneJET RNA purification kit, Thermo Scientific, USA) and cDNA synthesis (High Capacity cDNA Reverse Transcription Kit, Applied Biosystem, USA) for quantitative expression of cytokines. The expression of the following cytokines: IL1β, IL4, IL17, TNFα, IFNγ and anti-inflammatory cytokines: IL10, TGFβ. Experiments were carried out using the 7500 Fast Real-Time PCR System (Applied Biosystems, Singapore) and the Maxima SYBR Green Master Mix (Thermo Scientific, USA). The 2^−ΔΔct^ method was used to reflect the relative expression of mRNA, and GAPDH was used as a reference gene. Amplification protocol involved an initial denaturation of 95°C for 10 min, followed by 40 cycles at 95°C for 15 sec and 60°C for 30 sec.

### Statistical analysis

Statistical analyses were carried out using GraphPad Prism 8.4 (GraphPad, La Jolla, CA, USA). All experiments were performed in triplicate and repeated three independent times. Data were expressed as mean ± standard deviation or standard error, as indicated. Comparison between two groups was carried out using the two-tailed unpaired student’s *t*-test and Mann-Whitney test. The differencewas considered to be statistically significant when *P* < 0.05.

## Results

### S. haemolyticus *isolates exhibited variable hemolytic activities*

**Figure 1.**
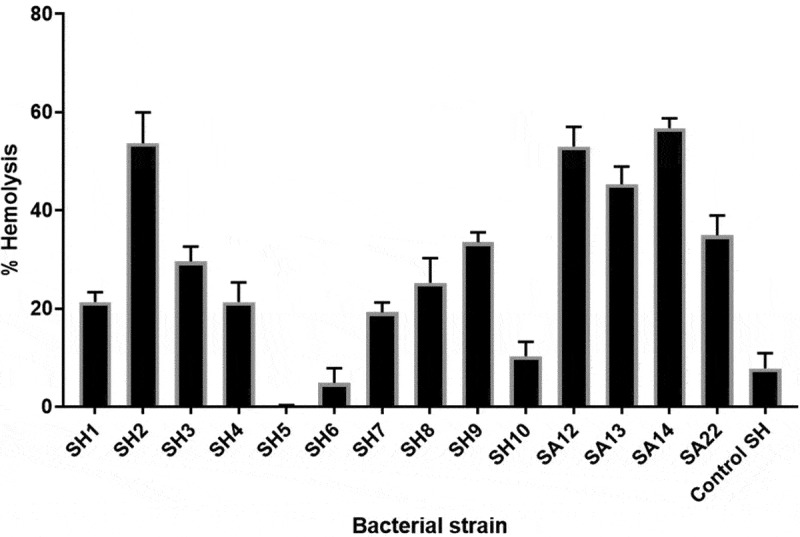
Hemolytic activities ofclinical *S. haemolyticus* isolates compared to *S. aureus* and control*S. haemolyticus* (ATCC 29970) after 5 h of incubation. SH; *S. haemolyticus*, SA; *S. aureus*. Depicted are the mean of 3 independent experiments ± standard deviation.

All *S. haemolyticus* isolates induced lysis of the RBCs, but with different extents. The hemolytic activities ranged between 0.1% and 53.7% (Figure 1). The highest activity was observed for strain SH2 (53.7%), which induced hemolysis levels comparable to that of the *S. aureus* strains. Generaly, most of the clinical SH isolates had higher hemolytic activities compared to the control *S. haemolyticus* strain (ATCC 29970).

### S. haemolyticus *carry various virulence genes and can adhere to the PHSF cells*

**Figure 2.**
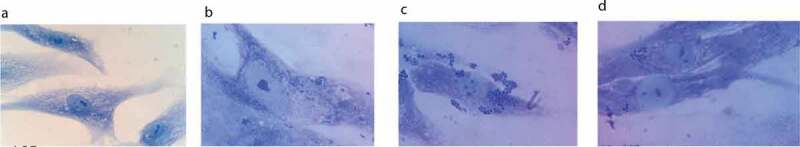
Bacterial adhesion to PHSF cells as detected by Giemsa stain. After 3 h in culture, PHSF cells were challenged with different *S. haemolyticus* isolates. (a) Non-infected fibroblast cells, (b) Fibroblasts infected with *S. haemolyticus* isolate with low adhesion capacity, (c) Fibroblasts infected with *S. haemolyticus* isolate with high adhesion capacity, (d) Fibroblasts infected with the control *S. haemolyticus* strain (ATCC 29970) showing low adhesion capacity.

*S. haemolyticus* isolates were evaluated for the presence of 12 virulence genesby PCR. Our data showed that each *S. haemolyticus* isolate carried at least two virulence genes (summarized in Table 2). Analysis of fibronectin-binding proteins showed that 9 out of 10 *S. haemolyticus* isolates possessed either *fnbA* or *fnbB* or both. *fnbB* gene was present in all strains except SH1, while *fnbA* gene was detected in 4 isolates. Both genes were present in 4 strains. *fnbA* and *fnbB* genes were absent in SH1. In addition, using Giemsastain, we tested the ability of *S. haemolyticus* to adhere to the PHSF cells (Table 2). The ability of the tested strains to adhere to the PHSF cells was variable. SH1 isolate showed the lowest adhesion capacity, while SH9 showed a high adhesion pattern (Figure 2(b-d)). Contrary to most clinical isolates, the control *S. haemolyticus* strain (ATCC 29970) showed a low adhesion capacity, with less than 100 adherent bacteria in 20 randomly selected microscopic fields. Interestingly, the SH1 clinical isolatethat showed the lowest adhesion ability, didn’t express *fnbA* or *fnbB* genes.

**Table 2.** Analysis of the different virulence-associated genes expressed by *S. haemolyticus.*

Strain	*Enterotoxins*							*Hemolysin*	*Exfoliative toxins*		*Fibronectin-binding protein*		Adhesion to PHSF cells*
	*sea*	*seb*	*sec*	*sed*	*see*	*seg*	*seh*	*hla*	*eta*	*etb*	*fnbA*	*fnbB*	
SH 1	-	-	+	-	-	-	+	+	-	-	-	-	+
SH 2	-	-	+	-	-	+	+	+	-	-	+	+	++++
SH 3	-	-	-	-	-	+	+	+	-	-	-	+	++
SH 4	-	-	-	-	-	-	-	+	-	-	-	+	++++
SH 5	+	-	-	-	-	-	-	-	-	-	-	+	++
SH 6	+	-	-	-	-	-	-	-	-	-	-	+	++++
SH 7	+	-	-	-	-	+	-	+	-	-	+	+	++
SH 8	-	-	-	-	-	-	-	+	-	-	-	+	+++
SH 9	-	-	+	-	-	+	-	+	-	-	+	+	++++
SH 10	+	-	+	+	-	+	-	+	-	-	+	+	++

*Adherent *S. haemolyticus* were countedmicroscopically (magnification x100) in 20 random microscopic fields and averaged. Very strong adhesion (++++) represented > 2500 adherent bacterial cells; strong adhesion (+++) represented 2500–1000 adherent bacteria; moderate adhesion (++) represented 1000–100 adherent bacteria and weak adhesion (+) represented less than 100 adherent bacteria.

We found that 8/10 of the *S. haemolyticus* isolates contained *enterotoxin* genes. Two strains contained one gene, 4 strains contained 2 genes, one strain contained 3 genes and one strain contained 4 genes. The most prevalent enterotoxin types were *seg* (5/10), *sea* (4/10) and *sec* (4/10). The *see* and *sed* genes were not detected. Most of the isolates (8/10) carried the *α-hemolysin* gene (6/10) (Table 2).

### Transmission electron microscopy (TEM)

**Figure 3.**
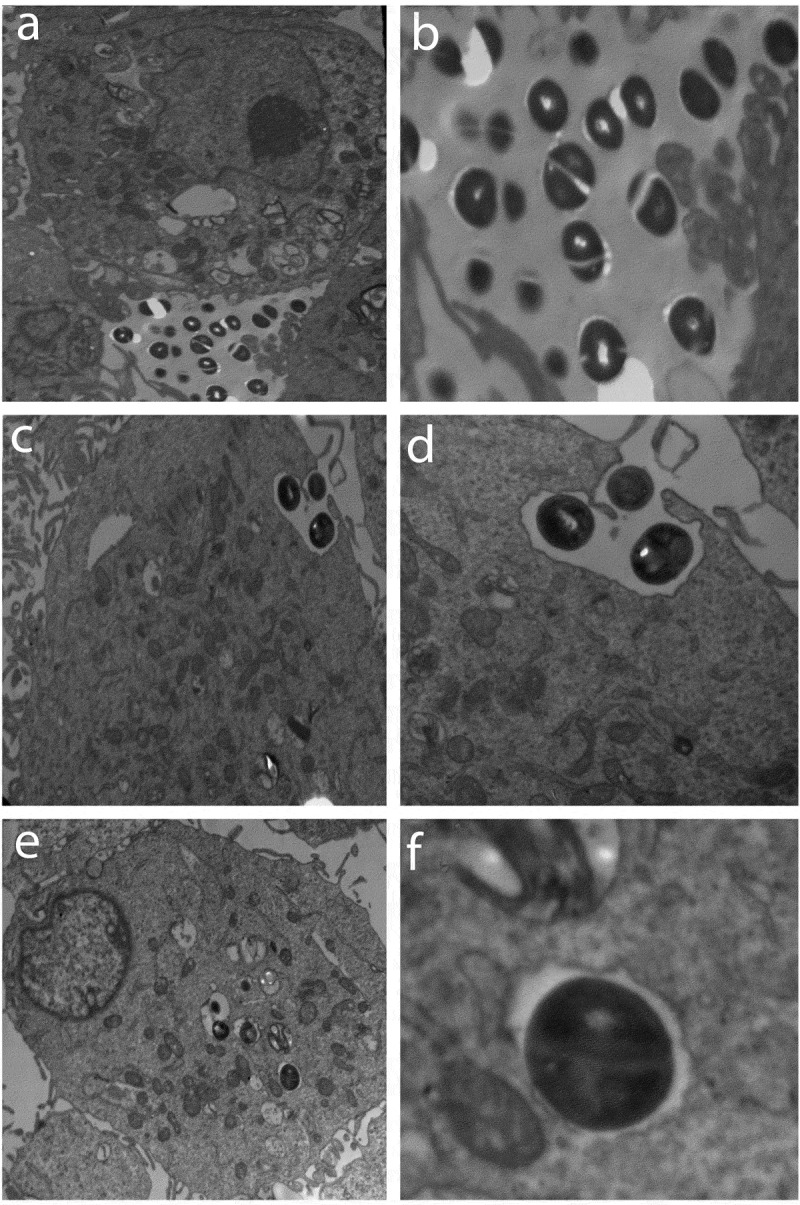
The PHSF cells were challenged with*S. haemolyticus* at an MOI of 10 and examined with TEM. (a) After 15 min of infection, extracellular bacteria were observed (3600x) which were further magnified (14,000 x) in (b). (c) After 1 h of infection, bacteria invaded the cells and became engulfed (3600 x). (d) Initialization of phagocytosis. (e) After 90 min, *S. haemolyticus* entered the PHSF cells and become localized in vacuoles (3600 x). (f) Proliferating bacteria are shown inside the vacuole.

To visualize the invasion of cultured PHSF cells by *S. haemolyticus*, monolayers of infected PHSF cells were examinedby TEM at 15, 60 and 90 min post-infection. After 15 min of infection, most of the bacteria were observed extracellularly (Figure 3(a,b)). However, after 1 hpseudopodia, engulfing the bacteria in a zipper-like mechanism could be detected (Figure 3(c,d)). After 90 min, *S. haemolyticus* was engulfed within a vacuole inside the cell, pointing to the ability of *S. haemolyticus* to enter, survive and proliferate inside thePHSF cells (Figure 3(e, f)).

### Flow cytometric invasion assay

**Figure 4.**
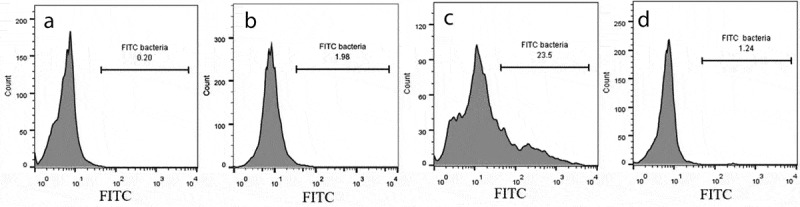
Invasion of PHSF cells by FITC-labeled bacteria. Comparative histograms represent results of flow cytometry analysis of (a) control non-infected cells (mock), (b) poorly invasive strain of *S. haemolyticus* (SH1), (c) highly invasive strain of *S. haemolyticus* (SH10), (d) Control *S. haemolyticus* strain (ATCC 29970). The figure shows a representative of three independent experiments.

To confirm that bacterial internalization takes place by the different isolates, PHSF cells were incubated with different FITC-labeled *S. haemolyticus*strains and the frequency of PHSF cells that acquired the FITC-labeled cells was recorded at 3 h post infection with flowcytometry. The percentage of internalized bacteria was variable. SH1 again showed a lower invasion rate compared to SH2, which demonstrated a higher invasion rate (Figure 4). Again, the clinical isolates had superior cellular invasion abilities compared to the control SH strain.Summary of the PHSF FITC^+^ cells that are infected with different isolatesis shown in Table 3.

**Table 3.** Summary of the frequency of virulence genes and invasion efficiencies of the*S. haemolyticus* strains.

Bacterial strain	Number of virulence genes	Frequency of FITC^+^ PHSF cells	Percentage of internalized bacteria relative to added cells±SD
SH 1	3	0.91	0.1 ± 0.2
SH 2	6	23.5	2 ± 0.3
SH 3	4	21.0	1.5 ± 0.35
SH 4	2	20.4	1.55 ± 0.15
SH 5	2	21.3	1.6 ± 0.08
SH 6	2	22.5	1.6 ± 0.07
SH 7	5	24.7	2.05 ± 0.09
SH 8	2	23.4	1.65 ± 0.09
SH 9	5	25.0	1.6 ± 0.11
SH10	7	26.9	2.5 ± 0.07
Control SH	ND*	1.24	0.25 ± 0.1

ND; Not determined

### S. haemolyticus *efficiently invade the PHSF cells*

**Figure 5.**
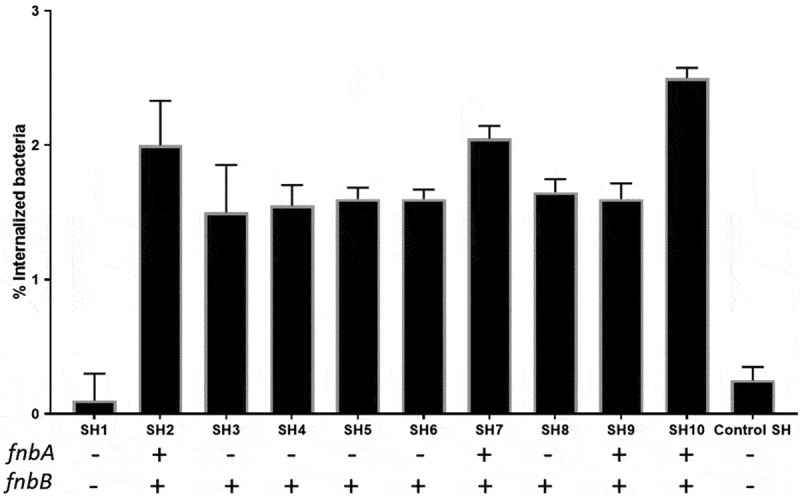
Percentage of internalized*S. haemolyticus* in the PHSF cells and the distribution of the *fnBP* genes.

We next aimed to quantify the CFU that invaded the PHFS cells. To do that, we challenged the PHSF cells with the *S. haemolyticus* strains for 3 h and then treated the cells with lysostaphin to lyse the extracellular or adherent staphylococci. Most *S. haemolyticus* strains effectively invaded thePHSF cells and achieved high intracellular levels. Of note, SH1 which was negative for both *fnbA* and *fnbB*, showed a low invasion capacity (Figure 5). SH2, SH7, SH9 and SH10 carried both the *fnbA* and *fnbB* genes and showed a higher level of invasion compared to other isolates. Mean percentage of internalized bacteria = 1.56 ± 0.06 and 2 ± 0.36 in case of cells infected with bacteria that carried *fnbB* only or infected with bacteria that carried both *fnbA* and *fnbB* genes, respectively (p value = 0.027 calculated with unpaired *t-*test). The *fnBP* genes are probably not the only determinants of the invasion capabilities of the *S. haemolyticus* tothe PHSF cells because SH1 strain, which did not carry the *fnbA* or *fnbB* gene was also able to invade and enter the cells although to a lower level. Of note, some strains carried a low number of virulence genes but were still able to invade the skin fibroblasts to remarkable levels (e.g SH4, SH5, SH6 and SH8) (Table 3).

### S. haemolyticus *and their cell-free supernatants affect the proliferation rate of the PHSF cells*

Since*S. haemolyticus* was able to invade the PHSF cells, we wondered whether *S. haemolyticus* could affect the viability of the infected cells. Therefore, the MTT assay was performed to study the effect of *S. haemolyticus* or its products on the viability of PHSF cells. *S. aureus* and control *S. haemolyticus* were used for comparison purposes. The capacity of bacterial suspension, cell-free supernatants, or heated bacteria to impair the viability of PHSF cells was determined at 6, 18, and 24 h post-infection. Our results showed that bacterial suspensions and cell-free supernatants of the clinical *S. haemolyticus* strains exhibited high cytotoxicity compared to the control *S. haemolyticus* strain.However, this cytotoxic effect was inhibited when cells were heated at 65°C for 30 min.

**Figure 6.**
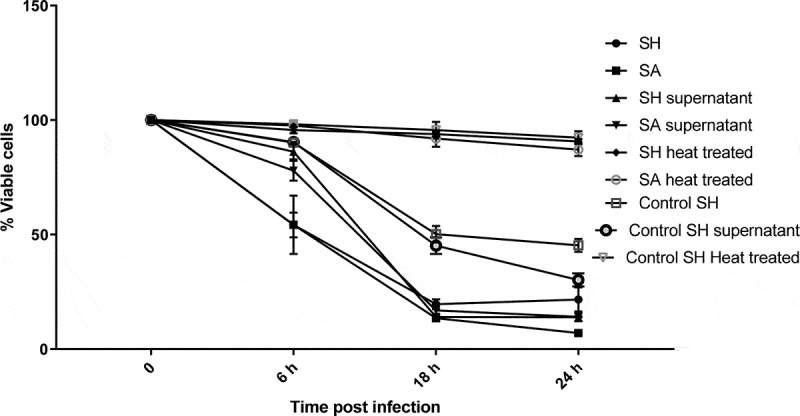
Effect of *Staphylococci* on the proliferation rate of PHSF cells measured by MTT assay. Data are represented as the mean of 3 experiments ± standard error.

The decrease in PHSF viability occurred in a time-dependent manner. At approximately only 6 h after adding the bacteria or the filtered supernatants,the percentage of viable cells was markedly reduced, and reached a dramatic level after 18 h (Figure 6). Generally, the effects of the bacterial suspensionsand the cell-free supernatants of *S. haemolyticus* and *S. aureus* were similar. The mean percentages of viable PHSF cells infected with whole *S. haemolyticus* suspensions were 54.2%±5.4, 19.6%±2.1 and 21.7%±5.2 at 6, 18 and 24 hpost-infection, respectively. Similarly, the mean percentage of viable PHSF cells infected with whole *S. aureus* isolates were 54.3%±12.7, 13.5%±1.2 and 7.0%±0.7 after 6, 18 and 24 h of infection. When cells were treated with *S. haemolyticus* supernatants, 86.2%±3.4, 14.0%±1.4 and 13.9%±1.7 were viable after 6, 18 and 24 h of infection, respectively. The values were comparable to those obtained when cells were treated with *S. aureus* supernatants (77.9%±4.3, 16.9%±2.8 and 14.1%±0.3 after 6, 18 and 24 h of infection, respectively).

### S. haemolyticus *and their cell-free supernatants induce apoptosis in the PHSF cells*

**Figure 7.**
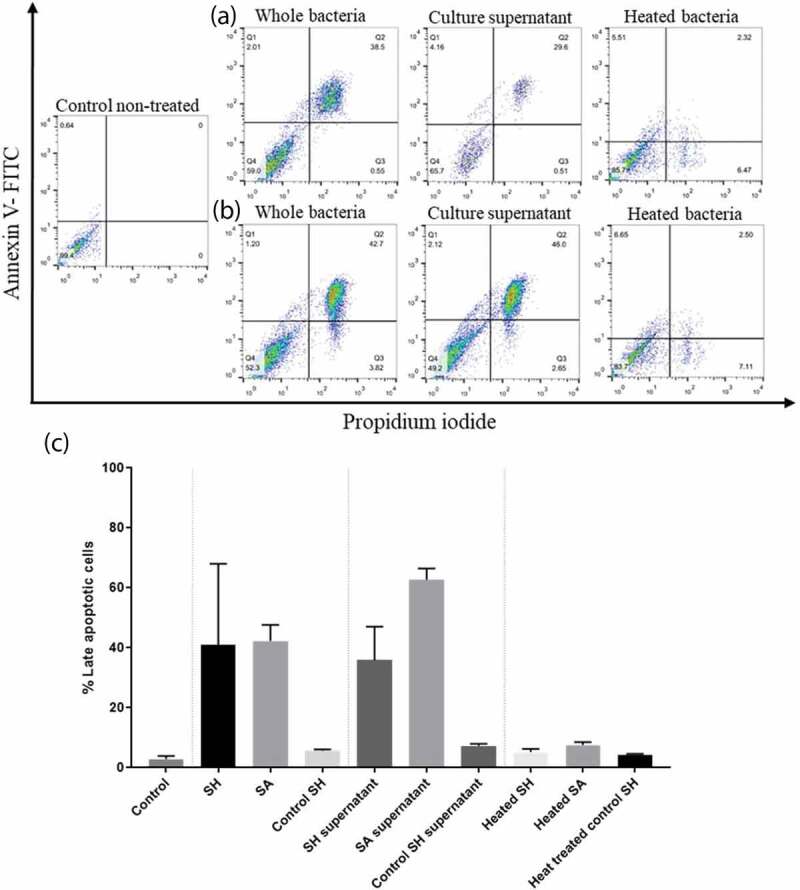
Flow cytometry analysis showing the cytotoxic effect of SH and SA on the PHSF cells. Cells were treated with indicated microbial products and stained after 24 h of incubation with Annexin V and PI. (a) Cells were infected with SH (upper panel), while in (b) cells were infected with SA (lower panel). (c) The mean of the cytotoxic activities of different tested strains on the PHSF cells ± SD. SH, *S. haemolyticus*; SA,*S. aureus.*

We determined whether clinical*S. haemolyticus* isolates can induce apoptosis in the PHSF cells. Flowcytometry using FITC-conjugated annexin V and PI staining revealed that PHSF cells exposed to the clinical strains of*S. haemolyticus* or *S. haemolyticus* supernatantsunderwent rapid apoptosis (Figure 7). The control *S. haemolyticus* (ATCC 29970) was unable to induce remarkable cytotoxicity in the PHSF cells. Similar results were observed when fibroblasts were challenged with the bacterial supernatant or when heat-treated. The mean percentage of apoptotic PHSF cells challenged with *S. haemolyticus* was highly similar to that obtained when cells were challenged with *S. aureus* (40.9%±15 and 42.3%±5.3, respectively, p-value > 0.05). However, more apoptotic cells were detected when PHSF cells were treated with SA supernatants (62.7% ± 3.75) than with SH supernatant (35.9%± 11.2) (p-value = 0.02). On the other hand, heat-treated SH and SA did not induce significant apoptosis.

### Analysis of cytokine expression

**Figure 8.**
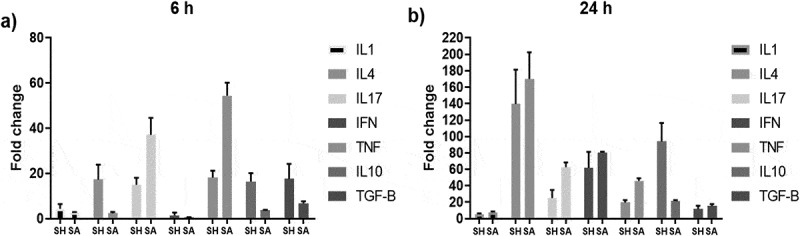
**Cytokine expression profile of PBMCs incubated with *S. haemolyticus* and *S. aureus* for 6 and 24 h**. The figure shows the mean of 3 independent experiments ± standard error.

The expression of different cytokines by PBMCs wasquantified after 6 and 24 hof co-culturing with*S. haemolyticus* or *S. aureus*. Both bacteria induced the expression of all tested cytokines after 6 h, which generally increased after 24 h (Figure 8). After 6 hof co-culture, *S. haemolyticus* induced higher levels of the inflammatory cytokines IL1β, IL4 and IFNγ compared to *S. aureus*. However, *S. haemolyticus* induced lower levels of the pro-inflammatory cytokines IL17 and TNFα. Also, higher levels of the anti-inflammatory cytokines IL10 and TGFβ were observed when cells were co-cultured with *S. haemolyticus*. While, after 24 h, *S. haemolyticus* induced lower levels of the pro-inflammatory cytokines IL1β, IL4, IL17, IFNγ, TNFα and the anti-inflammatory TGFβ compared to *S. aureus*. However, *S. haemolyticus* induced higher levels of the anti-inflammatory cytokines IL10.

## Discussion

Our study describedfor the first time the pathogenesis of *S. haemolyticus* isolated from patients with DFUs on PHSF cells.Staphylococci, including *S. haemolyticus*,arewidely distributed in hospital environment and are important causative agents of DFUs [23,24]. We employed an explant culture method for the isolation of the PHSF cells from human skin, which is a simple, reliable andinexpensive methodfor isolation of skin fibroblasts [25]. Keratinocytes did not attach to tissue culture plates and were removed by washing with PBSbecause keratinocytes need additional nutritional supplements and growth factors that were not included in our media [26].

### Detection ofthe virulence-related genes

The pathogenicity of *S. haemolyticus* isolates was directly associated with a broad spectrum of virulence factorswhich play a central role in the pathogenesis of staphylococcal infections. DFU is one of the leading causes of severe complications. These ulcers are caused by microorganisms that may carry different virulence factors. In this study, we analyzed a pool of *S. haemolyticus* isolates for the presence of genes encoding the enterotoxin, hemolysin, fibronectin-binding protein, and exfoliative toxins genes by PCR using pairs of specific primers.

Most *S. haemolyticus* isolates contained at least one type of the enterotoxin genes, and one isolate contained four different types. Staphylococcal enterotoxins (SE) constitute a family of nine major serological types of heat-stable enterotoxins that are biologically and structurally related. The most prevalent enterotoxin types were *seg, sea* and *sec* genes. Importantly, Skov, Olsen [27] reported that the application of the staphylococcal enterotoxin sebon human skin led to the induction of dermatitis, which may contribute to the damage induced by the infection of the diabetic foot ulcers. The ingestion of these toxins results in gastrointestinal manifestations such as nausea, vomiting, diarrhea, and abdominal pain. SEs are the leading cause of bacterial food poisoning in human beings and have been described as the cause of many outbreaks of foodborne diseases [28]. Moreover, these toxins are powerful superantigens that stimulate nonspecific T-cell proliferation and may lead to toxic shock syndrome. Secreted enterotoxins are the major weapons that kill host cells and cause diseases by inducing different types of cell death, particularly apoptosis and necrosis [29].

### Detection of the fibronectin-binding protein (FnBP) genes and adhesion assay

*Staphylococci* can express a variety of virulence factors, including surface proteins such as the FnBPs, which include *fnbA* and *fnbB*. These adhesive proteins play a crucial role in bacterial binding and adherence to the extracellular matrix and,accordingly, host cell invasion and bacterial internalization into cells [30–32]. Since keratinocytes and skin fibroblasts produce fibronectin and fibrinogen for the promotion of wound healing and tissue repair, these proteins will serve as a target or bacterial FnBPs [33]. Pereira, Teixeira [34]demonstrated that in *S. aureus*, increased virulence has been associated with its adhesive properties. Moreover, the *fnbB* genes were also involved in intercellular accumulation and development of biofilms [35]. Microbial infections resulting from bacterial adhesion to biomaterial surfaces have been observed on almost all medical devices [36].

We found that most *S. haemolyticus* isolates possessed either *fnbA* or *fnbB* or both. *fnbA* and *fnbB* genes were absent in SH1. Interestingly, this strain had low ability of invasion into the PHSF cells, pointing to the vital role of *fnbA* and *fnbB* genes in adhesion to host cells. Similar to our observation, Tuchscherr, Korpos [5] showed that the lack of human umbilical vein endothelial cells (HUVECs) invasiveness in *S. aureus* isolates was due to defects in FnBPs. The *fnBP* genes are commonly detected in *S. aureus* and different researchers reported variable percentages of the *fnbA* and *fnbB* genes. Tristan, Ying [37] demonstratedthat 43% of *S.aureus* were positive for *fnbB* and 28% were positive for *fnbA*. In 72 Tunisian MRSA isolates, *fnbA* gene was detected in 12 strains, *fnbB* gene in 2 strains, and both *fnbA* and *fnbB* genes in 2 other strains [38]. Also, Mirzaee, Najar-Peerayeh [39] showed that the prevalence of *fnbA* and *fnbB* in MRSA strains was 82.2% and 46.7%, respectively.

Although fibronectin adhesion is a major explanation for *S. aureus* virulence, CoNS internalization is a more controversial issue [40]. Interestingly, Switalski, Ryden [41] showed that *S. haemolyticus* isolates were able to bind fibronectin. Moreover, *S. haemolyticus* could bind collagen and vitronectin in a time-dependent manner [42]. A recent study has examined the binding of *S. haemolyticus* to human keratinocytes and reported a better adherence of clinical *S. haemolyticus* compared to commensal strains. Surface shaving analysis identified 65 surface proteins, including 3 adhesins, extracellular matrix binding protein (Embp), Mannosylglucosyl-3-phosphoglycerate phosphatase (SasH-like) and others. Upon colonization, an increase in *SceD* and the autolysin *Atl* genes was observed [43]. In fact, no previous reports have clearly detected FnBP proteins inCoNS [44]. Therefore, our results propose a new mechanism for adhesion and internalization of *S. haemolyticus*. However, more experiments are required to test the expression and upregulation of these genes upon infection of the cells. *S. epidermidis* has been shown to bind fibronectin bythe extracellular matrix binding protein (Embp), which facilities adhesion to fibronectin and formation of biofilm layers. One of the proposed explanations is that *S. epidermidis* enters the cells through a tripartite Embp-Fn-α5β1 system which is analogous to the FnBP-Fn-α5β1 integrin in *S. aureus* [45]. Indeed, Campoccia, Testoni [46] has found that *S. epidermidis* internalization was 100 times lower than *S. aureus* internalization.

### *Invasive of the PHSF cells by* S. haemolyticus

The ability of *S. aureus* to be internalized by host cells is considered one of the most critical pathogenicity factors in persisting and relapsing infections [44]. Trends of invasion and intracellular survival were similar in flowcytometry and lysostaphin protection experiments. All *S. haemolyticus* isolates could invade the PHSF cells to varying degrees. It seems that fibronectin-binding proteins of *S. haemolyticus* are essential in the process of internalization by PHSF cells since SH1, which was deficient in both genes, produced the least ability of adhesion and invasion. However, the presence of at least one of the known FnBP was significantly associated with marked adhesion and invasion of PHSF cells. Strains that carry both genesinvaded the PHSF cells at a significantly higher level than the other strains. However, more studies are required to test and to quantitatively analyze the expression of the *FnBP* upon infection of the PHSF cells by *S. haemolyticus*.

### Cytotoxicity and apoptosis

The effect of *S. haemolyticus* on the viability of PHSF cells was tested by observing two outcomes; the reduction of cell viability and proliferation measured by MTT assay and induction of apoptosis, which was measured by Annexin V/PI staining using flow cytometry. We tested whether the cytotoxic effects were caused by secreted factors or caused only by direct cell invasion. The bacterial cells caused cytotoxic effects similar to those obtained by the cell-free supernatants. This means that viablemetabolically active bacteria were not necessary for the cell-killing ability of the tested isolates and that the bacterial products induced cytotoxic effects, which were comparable to that induced by cellular invasion. The effects on cell viability and apoptosis induced by *S. haemolyticus* were comparable to the levels induced by *S. aureus* strains. However, the induction of cytotoxicity was lost when cells were incubated with heat-treated bacteria (65°C for 30 min), indicating the heat-labile properties of the toxic bacterial products.

The cytotoxic effect induced by bacteria is highly dependent on the virulence of the tested microorganism and the cell line [47]. This study was the first report that described the cytotoxicity of *S. haemolyticus* bacteria on PHSF cells. In our study, *S. haemolyticus* isolates exhibited different levels of cytotoxic activity ranging from 11.2% to 80.4%. The cytotoxic activity of whole *S. haemolyticus* and cell-free supernatants were similar to that induced by whole *S. aureus* and their supernatants. *S. haemolyticus* and the filtered bacterial supernatant remarkably decreased proliferation and increased apoptosis of the PHSF cells, with different degrees. In another study, *S. haemolyticus* was able to induce cytotoxicity of the HEp-2 cells (human epidermoid carcinoma cells from the larynx) that ranged from 13.8 to 81.5%. In the same study, preheating of the cell-free supernatants at 56 °C reduced the cytotoxic activity from 7.1% ±1.7% to 19.6% ±2.9% [18]. Moreover, Krzyminska, Szczuka [48] showed that all strains of *S. haemolyticus* exhibited cytotoxic effects on murine macrophage cell line J774, which were evident by the detachment of the cells from the surface of the wells. They also reported that three strains (10%) showed low cytotoxic activity.

In contrast to our results, Johansson, Rautelin [49] demonstrated that *S. aureus* isolates were not cytotoxic to HeLaor HT29 cells. Also, El-Housseiny, Aboulwafa [50] showed that the cell-free supernatants of the four *E. coli* isolates caused nearly no cytotoxicity after 3 h of Vero cell infection. On the other hand, the washed bacterial cells caused high cytotoxic effects similar to those obtained by the whole culture. In another study, the filtered supernatant from *S. aureus* failed to induce apoptosis when applied to endothelial cell monolayers, thereby excluding any effect from the preformed α-toxin or other soluble bacterial factors [51]. Also, van Kruchten, Wilden [52] showed that human alveolar basal epithelial cell line (A549), bronchial epithelial cell line (Calu-3), primary human umbilical vein endothelial cells (HUVECs) and human bronchial epithelial cells (HBEpCs) infected with *S. aureus* alone displayed no apoptosis. Heat treating of *S. haemolyticus* markedly interfered with the bacterial cytotoxic potential on the PHSF cells. In line with our results, Kahl, Goulian [53] showed that apoptosis induced in epithelial cells by infection with heat-killed *S. aureus* did not manifest any DNA fragmentation. Contrary to our results, Ocana, Asensi [54] showed that theinduction of apoptosis in neutrophil incubated with bacteria did not require live bacteria since organisms killed by heat treatment induced the same apoptotic effect.

### *Detection of hemolysin and* S. haemolyticus *hemolytic activity*

Our results showed that different levels of hemolytic activities were detected in all *Staphylococcus* isolates. It is generally considered that *S. aureus* produces four types of hemolysins (alpha, beta, gamma, and delta), which have hemolytic and cytotoxic effects. In particular, the *α-hemolysin* (*hla*) gene causes significant hemolysis [55]. Moreover, *hla* of *S. aureus* was shown to induce apoptosis in peripheral T lymphocytes [56]. The toxin also disrupts the tissue barrier at host interfaces lined by epithelial or endothelial cells [57]. Seidl, Leemann [58] showed that *S. aureus* isolates with α-toxin production induced significantly more endothelial damage compared to isolates without α-toxin production.Our results are in agreement with others who reported that *hla* is the most common *hemolysin* gene in *Staphylococci*. Moraveji, Tabatabaei [59] reported that *hla* is more frequently expressed by *S. haemolyticus* isolated from ulcers of diabetic patients. Pinheiro, Brito [60] showed that the *hla* gene was present in 91.7% of *S. haemolyticus* isolates. Also, Alfatemi, Motamedifar [61] showed that 93.15% *S. aureus* isolates were positive for the *hla* gene.

### *Impact of* S.haemolytics *infection on cytokine expression*

In order to improve our knowledge of the interaction between the immune system and *S. haemolyticus* during the infection, we investigated the expression of IL1β, IL4, IL17, IFNγ, TNFα, TGFβ and IL10 by PBMCs infected with*S. haemolyticus. S. haemolyticus* and *S. aureus* induced human PBMCs to produce different cytokines at different relative levels after 6 and 24 hof infection.We observed a marked early inflammatory response, which is consistent with other reports that described a similar production of high levels of inflammatory mediators (IL1β, IL6, and TNFα) after *S. aureus* peritoneal infection [62]. This could be explained by the expression of different types of Toll-like receptors on PBMCs, which interacts with the bacteria and its products, leading to an increase in inflammatory cytokine release [63]. Similar to our observation, stimulation of the keratinocytes with *S. epidermidis* evoked the gene expression and release of the powerful pro-inflammatory IL1β beta [64]. IL17 plays a key role in the defense of the host against different pathogens, including bacteria and viruses [65]. IL17 also produces a synergistic effect with other cytokines, such as IL1, IL6 and TNFα, to enhance the ability of the tissue infiltrating neutrophils to clear the extracellular pathogens [66]. Islander, Andersson [67] reported that superantigens produced by *S. aureus* were efficient in stimulating IL17 release. According to our results, IL4 expression was greater than IL1. Giese, Sumner [68] reported that IL4 might induce inhibitory effects on IL1β production, resulting in a reduced level.

Staphylococci also induced a marked increase in the expression of the down regulatory IL10 cytokine, particularly after 24 h, which attenuated the inflammatory response and may aid in improving the skin swelling, erythema and inflammation [69]. The peptidoglycan in the cell wall of Staphylococci induces the release of known immunosuppressive mediators such as IL10, PGE_2_, and TGFβ [1]. IL10 may play a protective role by inhibiting Th1 responses and blocking the expression of the pro-inflammatory cytokines [70]. Since IL10 is known to inhibit the synthesis of IFNγ as well as IL1, IL6, IL8 and TNFα. IL10 may dampen the inflammation and cytotoxic effect of these cytokines, which prevents chronic inflammation and host morbidity. On the other hand, the attenuation of the immune responses may lead to an increase in bacterialload [71,72]. We noticed that TNF levels did not increase further after 24 h of infection compared to their levels after 6 h. It is possible that the marked increase in IL10 levels hindered the further increase in TNF expression levels.

We concluded that *S. haemolyticus* carries an inclusive set of genes that code for different virulence factors such as toxins, enzymes and adhesion proteins. Our results contribute to a better understanding of the pathomechanisms of *S. haemolyticus* infections and fill a gap in the literature regarding the CoNS. Based on our observations, microbiology laboratories should consider *S. haemolyticus* as a critical opportunistic pathogen and infections caused by this organism, particularly in critically ill patients, should be seriously managed. Additional experiments are planned to study the expression of these genes upon infection of the PHSF cells and the role of these genes in the invasion, intracellular survival and establishment of infection in the host cells. These factors may contribute to the bacteria’s ability to spread through tissues and the pathological damage associated with DFU. Among the limitations of this study is the low number of tested isolates and that the all tested bacteria were obtained from only one site of infection. However, in future studies, we are planning to test the pathogenesis of more clinical strains that are collected from different sites of infection.

## Supplementary Material

Supplemental Material
