# Supplementary material for: Pathogenesis of Staphylococcus haemolyticus on primary human skin fibroblast cells
Source: Virulence. 2020 Aug 30;11(1):1142–57. doi: 10.1080/21505594.2020.1809962 (PMC7549902; doi:10.1080/21505594.2020.1809962)
Supplement: Supplemental Material [file KVIR_A_1809962_SM1133.docx]

**Supplemental Table 1:**

**Table S1: Characteristics of the diabetic patients and the antimicrobial resistance profile of the isolated *S. haemolyticus* used in the study**

| **Patient ID** | **Gender** | **Age (years)** | **Date of isolation** | **Infected site within the leg** | **Resistant antibiotics** | **Sensitive antibiotics** |
| --- | --- | --- | --- | --- | --- | --- |
| Patient #1 | Male | 67 | 6^th^ -March 2018 | Metatarsal head | ceftriaxone, cefoxitin, ciprofloxacin, amikacin, ceftazidime, chloramphenicol, tetracycline, tobramycin, oxacillin, amoxicillin/clavulanic acid, Erythromycin | vancomycin, linezolid, rifampicin, clindamycin, Trimethoprim/sulfamethoxazole |
| Patient #2 | Female | 52 | 9^th^ - May 2018 | Big toe | ceftriaxone, cefoxitin, ciprofloxacin, clindamycin, amikacin, ceftazidime, chloramphenicol, tetracycline, tobramycin, Trimethoprim/sulfamethoxazole, oxacillin, amoxicillin/clavulanic acid, Erythromycin | vancomycin, linezolid |
| Patient #3 | Male | 46 | 12^th^ - June 2018 | Forefoot | ceftriaxone, cefoxitin, ciprofloxacin, amikacin, ceftazidime, tobramycin, Trimethoprim/sulfamethoxazole, oxacillin, amoxicillin/clavulanic acid, Erythromycin | vancomycin, linezolid, clindamycin, chloramphenicol, tetracycline, |
| Patient #4 | Male | 55 | 25^th^ - June 2018 | Big toe | ceftriaxone, cefoxitin, ciprofloxacin, clindamycin, amikacin, ceftazidime, chloramphenicol, tetracycline, tobramycin, Trimethoprim/sulfamethoxazole, oxacillin, amoxicillin/clavulanic acid | vancomycin, linezolid, rifampicin, Erythromycin |
| Patient #5 | Male | 48 | 16^th^ - Juli  2018 | Metatarsal head | cefoxitin, ciprofloxacin, clindamycin, amikacin, Trimethoprim/sulfamethoxazole, oxacillin, amoxicillin/clavulanic acid, Erythromycin | vancomycin, linezolid, Ceftriaxone, tobramycin, chloramphenicol, tetracycline, ceftazidime, |
| Patient #6 | Male | 62 | 23^th^ - August 2018 | Forefoot | cefoxitin, ciprofloxacin, clindamycin, amikacin, ceftazidime, chloramphenicol, tetracycline, tobramycin, Trimethoprim/sulfamethoxazole, oxacillin, amoxicillin/clavulanic acid | vancomycin, linezolid, ceftriaxone, erythromycin |
| Patient #7 | Female | 66 | 29^th^ - August 2018 | Metatarsal head | cefoxitin, ciprofloxacin, clindamycin, amikacin, ceftazidime, chloramphenicol, tetracycline, tobramycin, Trimethoprim/sulfamethoxazole, oxacillin, amoxicillin/clavulanic acid, erythromycin | vancomycin, linezolid, , rifampicin, ceftriaxone, |
| Patient #8 | Female | 59 | 19^th^ -January 2019 | Metatarsal head | ceftriaxone, cefoxitin, ciprofloxacin, clindamycin, amikacin, ceftazidime, chloramphenicol, tetracycline, tobramycin, Trimethoprim/sulfamethoxazole, oxacillin, amoxicillin/clavulanic acid, erythromycin | vancomycin, linezolid, |
| Patient #9 | Male | 54 | 6^th^ - February 2019 | Metatarsal head | ceftriaxone, cefoxitin, ciprofloxacin, clindamycin, amikacin, ceftazidime, chloramphenicol, tetracycline, tobramycin, Trimethoprim/sulfamethoxazole, oxacillin, amoxicillin/clavulanic acid, erythromycin | vancomycin, linezolid, |
| Patient #10 | Female | 63 | 11^th^ - February 2019 | Metatarsal head | ceftriaxone, cefoxitin, ciprofloxacin, clindamycin, amikacin, ceftazidime, chloramphenicol, tetracycline, tobramycin, Trimethoprim/sulfamethoxazole, oxacillin, amoxicillin/clavulanic acid, erythromycin | vancomycin, linezolid, |

**Title page:**

Pathogenesis of *Staphylococcus haemolyticus* on primary human skin fibroblast cells

Hala O. Eltwisy^1^, Medhat Abdel-Fattah^2^, Amani M. Elsisi^3^, Mahmoud M. Omar^4^, Ahmed Aly Abdelmoteleb^5^, Mohamed A. El-Mokhtar^6,*^

1 Department of Microbiology, Faculty of Science, Beni-Suef University, Egypt.

2 Department of Microbiology and Botany, Faculty of Science, Beni-Suef University, Egypt

3 Department of Pharmaceutics and Industrial Pharmacy, Beni-Suef University, Egypt

4 Department of Pharmaceutics and Industrial Pharmacy, Deraya University, El-Minia, Egypt

5 Department of General Surgery, Faculty of Medicine, Assiut University, Egypt

6 Department of Medical Microbiology and Immunology, Faculty of Medicine, Assiut University, Egypt

^*^**Corresponding author**

Ass. Prof. Mohamed A. El-Mokhtar

Department of Medical Microbiology and Immunology,

Faculty of Medicine, Assiut University, Egypt, 71515.

Tel: 002-0122 111 53 13

Fax: 088-2332278-2080278

Email: [elmokhtarma@aun.edu.eg](mailto:elmokhtarma@aun.edu.eg) [ma_mokhtar@yahoo.com](mailto:ma_mokhtar@yahoo.com)

**Abstract**

*Staphylococcus haemolyticus* (*S.haemolyticus*) is one of the Coagulase-negative-staphylococci (CoNS) that inhabits the skin as a commensal. It is increasingly implicated in opportunistic infections, including diabetic foot ulcer (DFU) infections. In contrast to the abundance of information available for *S. aureus* and *S. epidermidis*, little is known about the pathogenicity of *S. haemolyticus*, despite the increased prevalence of this pathogen in hospitalized patients. We described, for the first time, the pathogenesis of ten different clinical isolates of *S. haemolyticus* isolated from DFU on primary human skin fibroblast (PHSF) cells. Virulence-related genes were investigated, adhesion and invasion assays were carried out using Giemsa stain, transmission electron microscopy (TEM), MTT and flow cytometry assays. Our results showed that most *S. haemolyticus* carried different sets of virulence-related genes. *S. haemolyticus* adhered to the PHSF cells to variable degrees. TEM showed that the bacteria were engulfed in a zipper-like mechanism into a vacuole inside the cell. Bacterial internalization was confirmed using flow cytometry and achieved high intracellular levels. PHSF cells infected with *S. haemolyticus* suffered from a marked decrease in viability and increased apoptosis when treated with whole bacterial suspensions or cell-free supernatants but not with heat-treated cells. After co-culture with PBMCs, *S. haemolyticus* induced high levels of pro-inflammatory cytokines*.* This study highlights the great development of *S. haemolyticus,* which was previously considered a contaminant when detected in cultures of clinical samples. Their high ability to adhere, invade and kill the PHSF cells illustrate the severe damage associated with DFU infections.

**Keywords**: *Staphylococcus haemolyticus*; Diabetic foot ulcer; Primary human skin fibroblast cells; bacterial invasion; Pathogenesis.

**Abbreviations**: CoNS, coagulase-negative staphylococci; DFU, diabetic foot ulcer; DM, diabetes mellitus; DMEM, Dulbecco's Modified Eagle Medium; MTT, 3-(4, 5-dimethylthiazolyl-2)-2, 5-diphenyltetrazolium bromide; PBMCs, peripheral blood mononuclear cells; PHSF, primary human skin fibroblast; CFU, colony-forming unit.

**Introduction**

*Staphylococcus haemolyticus* (*S. haemolyticus*) is one of the coagulase-negative staphylococci (CoNS) that inhabit the skin as a commensal. It is increasingly implicated in opportunistic infections in immunocompromised patients, particularly in hospitalized patients and those with medical implants worldwide [[1](#_ENREF_1)]. It is the second most frequent CoNS isolated from infected clinical samples, particularly blood cultures of patients with sepsis following *S. epidermidis* [[2](#_ENREF_2)].

*S. haemolyticus* causes severe infections in several body systems including meningitis, endocarditis, prosthetic joint infections and bacteremia and is prevalent in the hospital environment and on the hands of healthcare workers. *S. haemolyticus* is also known to cause septicemia, peritonitis, otitis media and diabetic foot ulcer (DFU) infections [[3](#_ENREF_3), [4](#_ENREF_4)].

Diabetes mellitus (DM) is a metabolic disease that is associated with increased susceptibility to bacterial infections. Patients usually suffer from infected foot ulcers, which increases the complexity of their treatment. About 15% of patients with DM develop foot ulcers that may progress to osteomyelitis and amputation [[5](#_ENREF_5)]. These bacterial infections are usually caused by the coagulase-positive *Staphylococcus aureus* (*S. aureus*) and also the emerging CoNS, including *Staphylococcus epidermidis* (*S. epidermidis*) and *S. haemolyticus* [[6](#_ENREF_6)].

A characteristic feature of *S. haemolyticus* is its ability to form biofilms, which play an essential role in the establishment of infections. The produced exopolysaccharides can inhibit the growth of other bacteria and also decrease their ability to form biofilms [[7](#_ENREF_7)]. This species has gained an increased clinical significance due to its genome plasticity, which allowed a great adaptation and development of resistance to different antibiotics, including methicillin and its ability to survive in the hospital environment [[8](#_ENREF_8)]. The remarkable ability of *S. haemolyticus* to acquire antibiotic resistance, especially to oxacillin, limits the available therapeutic options for catheter-related infections caused by methicillin resistant *S. haemolyticus* isolates and may predispose to sepsis and increase patient’s morbidity and mortality [[9](#_ENREF_9), [10](#_ENREF_10)]. *S. haemolyticus* and *S. aureus* have >99.9% identities in the sequences of *beta-lactamase* and *qacA* genes, pointing to the possibility of interspaces exchange of the genetic elements responsible for resistance to antibiotics [[11](#_ENREF_11)].

In contrast to the abundance of information available for *S.* *aureus* and *S. epidermidis*, little is known about the pathogenicity and virulence factors of *S.* *haemolyticus*, despite the increased prevalence of this pathogen in immunocompromised patients [[12](#_ENREF_12)].

The aim of this study was to describe, for the first time, the pathogenesis of clinical isolates of *S. haemolyticus*, isolated from DFU on PHSF cells. Virulence-related genes were investigated, adhesion and invasion assays were carried out using Giemsa stain, transmission electron microscopy (TEM), MTT and flow cytometry assays. The potential cytotoxic and apoptotic effects induced by *S. haemolyticus* were investigated. Also, changes in cytokine profile in response to infection with *S. haemolyticus* were shown. Our results contribute to a better understanding of the pathomechanisms of *S. haemolyticus* infections and fill a gap in the literature regarding the CoNS. Based on our observations, microbiology laboratories should consider *S. haemolyticus* a critical opportunistic pathogen and infections caused by this organism, particularly in critically ill patients, should be seriously managed.

**Materials and methods**

**Ethics Statement**

The study protocol was approved by the Ethics Committee of the Faculty of Medicine, Assiut University (Assuit, Egypt) and conducted in accordance with the provisions of the Declaration of Helsinki (approval number 17300379). Informed written consent was obtained from study participants.

**Skin Punch Biopsy Explant Culture for preparation of the PHSF cells**

Isolation of PHSF cells from skin punch biopsy was performed as described before, with minor modifications [[13](#_ENREF_13), [14](#_ENREF_14)]. Skin biopsy specimens were obtained from anonymous healthy donors (aged between 20 and 35 years) during surgery procedures of abdominal dermolipectomy. Skin fragments were immersed in 70 % ethanol and washed 3 times by phosphate buffer saline (PBS). Fibroblast harvesting was done by explant technique. The dermis and epidermis were isolated from the subcutaneous tissue and fragmented into 5 mm^2^ pieces with scalpels and scissors. These fragments (epidermis upward and dermis downward) were laid onto the surface of a 6-well plate, which was pre-coated with 0.2% gelatin solution (Sigma, Germany). We used a coverslip to hold down the skin pieces that helped the adhesion of fragment to the gelatin-coated plates. DMEM supplemented with 30% (v/v) fetal bovine serum (FBS) and 1% (v/v) penicillin-streptomycin (all from Gibco, USA) were added to each well and medium was changed every 2-3 days. After 20 days, fibroblasts were sub-cultured using trypsin-EDTA (0.25%) (Gibco, USA).

**Bacterial isolates and species identification**

From March 2018 through February 2019, a prospective study of patients with diabetic foot ulcers admitted to the diabetic foot unit at Assiut University Hospital was conducted. One hundred patients were enrolled. After wound debridement, the wound base was swabbed using a cotton swab moistened with sterile 0.9% NaCl solution for bacterial culture, and samples were sent immediately to the microbiology department. Swabs were enriched into brain heart infusion broth (Becton Dickinson GmbH, Heidelberg, Germany) then streaked onto enriched and selective bacterial culture plates.

Staphylococci were identified using the VITEK2 Microbiology automated system (VITEK^®^2, BioMerieux). Moreover, the identity of staphylococci was confirmed using multiplex PCR as described previously [[15](#_ENREF_15)]. During the study period, ten S. haemolyticus isolates were obtained from the cultures of DFUs from ten different infected patients. In these infected ulcers, only S. haemolyticus was detected. We referred to these isolates as SH1 to SH10 in our infection experiments. In some experiments, four different S. aureus clinical isolates were used as controls and for comparison purposes (referred to as SA12, SA13, SA14 and SA22). These isolates were also recovered from patients suffering from severe deep DFU infections from the same hospital. In addition, *S. haemolyticus* (ATCC 29970) was used as a control in the phenotypic functional experiements (referred to in most experiments as control *S. haemolyticus*).

Methicillin resistance was detected by initial phenotypic screening for reduced susceptibility of the isolates to cefoxitin (30 µg) and oxacillin (1 μg) disks (Mast, UK) by the disk diffusion method, according to Clinical and Laboratory Standards Institute (CLSI) guidelines. Then, methicillin resistance was confirmed by amplification of the *mecA* gene by PCR assay using specific primers proposed by [Pinheiro, Brito [15]](#_ENREF_15).

**Hemolysis assay**

The hemolytic activity of *S. haemolyticus* isolates was analyzed according to the protocol of [Cremet, Broquet [16]](#_ENREF_16), using a clinical isolate of *S. aureus* as a control. Hemolytic activity was measured spectrophotometrically by using a microplate reader (Epoch™ Microplate Spectrophotometer, BioTek, USA). Briefly, the hemolytic activity of the strains was quantified in human blood diluted to 10% (v/v) in PBS. For these experiments, 200 μl of overnight bacterial suspension with a concentration of about 10^8^ CFU/ml in PBS was added to 200 μl of the erythrocyte suspension and the mixture was incubated for 5 h. Then samples were centrifuged at 1500 xg for 5 min. Hemoglobin release in the supernatant was observed by measuring absorbance at 450 nm. Blood treated with 1% Triton X-100 was used as a positive control (100% hemolysis) and blood treated with PBS was used as a negative control (0% hemolysis).

**Detection of enterotoxin, hemolysin, fibronectin binding protein and exfoliative genes**

PCR was used for the detection of different virulence-associated genes; *sea*, *seb*, *sec*, *sed*, *see*, *seg*, *seh*, *α-hemolysin*, *fnbA*, *fnbB*, *eta* and *etb*. Target genes and primer sequences are detailed in Table 1.

**Preparation of bacteria for infection experiments**

In our infection experiments, fresh bacterial suspensions were prepared according to the following protocol. Twenty-four hours prior to experiments, 2-3 colonies of *S. haemolyticus* were transferred from blood agar into LB broth and incubated at 37 °C. The overnight bacterial culture was centrifuged at 1500 xg for 5 min, the supernatant was removed and the cell pellet was suspended in 2 ml PBS. For preparation of bacterial supernatant, the supernatant of bacteria was filtered through 0.2 µm filter. For infection assays, PHSF cells were plated at a density of 10^5^ cells/wells in 12-well tissue culture plates with 1 ml of DMEM cell culture medium and 1% FBS without antibiotics (invasion medium). Before infection, cells were washed with PBS and the invasion medium was added and kept for 1 h at 37 °C. In all infection experiments, the optical density of bacteria was adjusted at OD_600_ = 0.2, which is equivalent to a multiplicity of infections (MOIs) of 10 bacteria per cell.

**Adhesion assay using Giemsa stain**

Adhesion of *S. haemolyticus* to PHSF cells was tested. To do that, washed bacterial suspension was added to cells on coverslips at MOI of 10, and cells were incubated for 3 h at 37 °C. Coverslips were washed 6 times to remove non-adhering bacteria by immersing the coverslips in saline. 500 μl of Giemsa stain (10%) was added to each well for 30–40 min at room temperature. Coverslips were washed to remove the excess of the stain or stain debris. Cells were fixed by adding 200 μl of 70% methanol for 5 min at -20 °C. Then, coverslips were washed by PBS to remove methanol and left to air dry. Coverslips were examined microscopically (magnification of x100) and the number of adherent bacteria was counted in 20 randomly selected microscopic fields and averaged. Very strong adhesion (++++) represented > 2500 adherent bacterial cells; strong adhesion (+++) represented 2500-1000 adherent bacteria; moderate adhesion (++) represented 1000 -100 adherent bacteria and weak adhesion (+) represented less than 100 adherent bacteria, similar to the method reported by [Guglielmetti, Taverniti [17]](#_ENREF_17).

**Transmission electron microscopy**

Electron microscopy was used to visualize the adhesion and invasion of *S. haemolyticus* into the PHSF cells. Cells were grown in T75 Flask and infected with *S. haemolyticus* as described above. After 15, 60 and 90 min post-infection, cells were washed three times with PBS and harvested by trypsinization. They were centrifuged at 1200 xg for 5 min, fixed with 2 % glutaraldehyde, washed two times with PBS and postfixed with 1 % OsO_4_ for 2 h. Cells were embedded in 2% agar for 15 min. Sample was dehydrated with sequential ethanol baths (30 to 100%) for 10 min, and embedded in Epon 812 resin with a 48 h polymerization time at 70 °C. By using an ultramicrotome, the embedded samples were sliced. Images were performed with a JEM 100 CX11 transmission electron microscope [[18](#_ENREF_18), [19](#_ENREF_19)].

**Flow cytometry invasion assay**

The ability of  *S. haemolyticus* to invade the PHSF cells was also analyzed by flow cytometry invasion assay as described elsewhere [[20](#_ENREF_20)], with minor modifications. Suspensions of FITC-labelled bacteria (OD_600_=0.2) were added to PHSF cells for 1 h at 4 °C to allow for sedimentation of bacteria and then changed to 37 °C for 3 h, to allow for cellular invasion. Finally, PHSF cells were harvested, and 100 μg/ml lysostaphin (Sigma-Aldrich, Germany) in PBS was added for 25 min at ambient temperature to remove extracellular bacteria. PHSF cells were transferred to 5 ml round-bottom polystyrene FACS tubes (Falcon; BD, Heidelberg, Germany), pelleted, and resuspended in PBS. Cells were analyzed by flow cytometry (BD FACSCalibur, USA) to determine the frequency of FITC-labelled cells.

**Lysostaphin protection assay**

To determine the colonization of PHSF cells by *S. haemolyticus*, we performed a lysostaphin protection assay [[21](#_ENREF_21)]. PHSF cells were infected with *S. haemolyticus* and incubated for 3 h in invasion medium. After three washes with PBS, lysostaphin (100 μg/ml) was added for 25 min. Cells were lysed by the addition of 1 ml 0.5% Triton X-100 (Sigma-Aldrich, Germany) in PBS for 20 min at 37 °C. Tenfold serial dilutions of cell lysates in sterile H_2_O were plated onto Nutrient Agar (Oxoid) and incubated overnight at 37 °C to quantify the intracellular bacteria by counting the colony-forming units (CFUs).

**Effect of *S. haemolyticus* on PHSF cell proliferation rate**

The effect of *S. haemolyticus* on the proliferation rate of PHSF cells was initially tested by using the MTT assay, as described by [Saliba, Filloux [22]](#_ENREF_22). Cells were seeded into 96-well plates at a density of 1 x 10^4^ cells/well. After 24 h, cells were treated with washed bacterial suspension, cell-free bacterial supernatant, or heated bacterial suspension. Heat killed bacteria were prepared by heating washed bacterial suspension at 65 ˚C for 30 min using the Heat block (Thermo-mixer, Germany). Control wells contained 100 μl DMEM, 50 μl sterile broth instead of the added bacterial culture. Plates were incubated at 37 °C, and cell viability was tested at different time points at 6, 18 and 24 h post-treatment. Cells were treated with 10 μl of 2 mg/ml MTT reagent (SERVA, Germany) in PBS for 4 h at 37 °C. 100 μl of DMSO (Sigma-Aldrich, Germany) was added per well to dissolve the formazan crystals formed in the viable metabolically active cells. The absorbance of the resultant solution was measured at 570 nm using a microplate Spectrophotometer (EPOCH, USA). The percentage of cytotoxicity was calculated using the following formula:

% Cytotoxicity = (100-$(\frac{OD570 of infected culture}{OD570 of control}$) X 100)

**Detection of apoptosis by flow cytometry**

To analyze the ability of *S. haemolyticus* to induce apoptosis in the PHSF cells, adherent PHSF cells were infected with washed *S. haemolyticus*. After 24 h, cells were de-attached by trypsin and apoptosis was detected by flow cytometry using Annexin V/PI staining (ApoFlowEx FITC Kit, exbio). Early apoptotic cells were stained with annexin V alone, whereas necrotic cells and late apoptotic cells stained with both annexin V and propidium iodide.

**Effect of** ***S. haemolyticus* on cytokine production**

Peripheral blood mononuclear cells (PBMCs) were separated from heparinized whole venous blood by density gradient centrifugation using Histopaque®-1077 (Sigma-Aldrich, Germany) following manufacturer’s instructions. PBMCs (1 x 10^7^ cells/ml) were suspended in RPMI 1640 medium (Gibco, USA) and infected with washed bacterial suspension of *S. haemolyticus* (MOI=10) in 6 well plates at 37 ˚C. PBMCs suspended in RPMI 1640 medium (Gibco, USA) without antibiotics served as a control to establish basal cytokine levels. After 6 and 24 h of incubation, cells were washed 2 times with PBS and incubated with lysostaphin then subjected to RNA extraction (GeneJET RNA purification kit, Thermo Scientific, USA) and cDNA synthesis (High Capacity cDNA Reverse Transcription Kit, Applied Biosystem, USA) for quantitative expression of cytokines. The expression of the following cytokines: IL1β, IL4, IL17, TNFα, IFNγ and anti-inflammatory cytokines: IL10, TGFβ. Experiments were carried out using the 7500 Fast Real-Time PCR System (Applied Biosystems, Singapore) and the Maxima SYBR Green Master Mix (Thermo Scientific, USA). The 2^-ΔΔct^ method was used to reflect the relative expression of mRNA, and GAPDH was used as a reference gene. Amplification protocol involved an initial denaturation of 95 °C for 10 min, followed by 40 cycles at 95 °C for 15 sec and 60 °C for 30 sec.

**Statistical analysis**

Statistical analyses were carried out using GraphPad Prism 8.4 (GraphPad, La Jolla, CA, USA). All experiments were performed in triplicate and repeated three independent times. Data were expressed as mean ± standard deviation or standard error, as indicated. Comparison between two groups was carried out using the two-tailed unpaired student’s *t*-test and Mann-Whitney test. The difference was considered to be statistically significant when *P* < 0.05.

**Results**

***S. haemolyticus* isolates exhibited variable hemolytic activities**

All *S. haemolyticus* isolates induced lysis of the RBCs, but with different extents*.* The hemolytic activities ranged between 0.1% and 53.7% (Figure 1). The highest activity was observed for strain SH2 (53.7%), which induced hemolysis levels comparable to that of the *S. aureus* strains. Generaly, most of the clinical SH isolates had higher haemolytic acitivity compared to the control *S. haemolyticus* strain (ATCC 29970).

***S. haemolyticus* carry various virulence genes and can adhere to the** **PHSF cells**

*S. haemolyticus* isolates were evaluated for the presence of 12 virulence genes by PCR. Our data showed that each *S. haemolyticus* isolate carried at least two virulence genes (summarized in table 2). Analysis of fibronectin-binding proteins showed that 9 out of 10 *S. haemolyticus* isolates possessed either fnbA or fnbB or both*.* *fnbB* gene was present in all strains except SH1, while *fnbA* gene was detected in 4 isolates. Both genes were present in 4 strains. fnbA and fnbB genes were absent in SH1. In addition, using Giemsa stain, we tested the ability of *S. haemolyticus* to adhere to the PHSF cells (Table 2). The ability of the tested strains to adhere to the PHSF cells was variable. SH1 isolate showed the lowest adhesion capacity, while SH9 showed a high adhesion pattern (Figure 2B-D). Contrary to most of the clinical isolates, the control *S. haemolyticus* strain (ATCC 29970) showed a low adhesion capacity, with less than 100 adherent bacteria in 20 randomly selected microscopic fields. Interestingly, the SH1 clinical isolate that showed the lowest adhesion ability, didn’t express *fnbA* or *fnbB* genes.

We found that 8/10 of the *S. haemolyticus* isolates contained *enterotoxin* genes. Two strains contained one gene, 4 strains contained 2 genes, one strain contained 3 genes and one strain contained 4 genes. The most prevalent enterotoxin types were seg (5/10), sea (4/10) and sec (4/10). The see and sed genes were not detected. Most of the isolates (8/10) carried the *α*-*hemolysin* gene (6/10) (Table 2).

**Transmission electron microscopy (TEM)**

To visualize the invasion of cultured PHSF cells by *S. haemolyticus*, monolayers of infected PHSF cells were examined by TEM at 15, 60 and 90 min post-infection. After 15 min of infection, most of the bacteria were observed extracellularly (Figure 3 A, B). However, after 1 h pseudopodia, engulfing the bacteria in a zipper-like mechanism could be detected (Figure 3 C, D). After 90 min, *S. haemolyticus* was engulfed within a vacuole inside the cell, pointing to the ability of *S. haemolyticus* to enter, survive and proliferate inside the PHSF cells (Figure 3 E, F).

**Flow cytometric invasion assay**

To confirm that bacterial internalization takes place by the different isolates, PHSF cells were incubated with different FITC-labelled *S. haemolyticus* strains and the frequency of PHSF cells that acquired the FITC-labelled cells was recorded at 3 h post infection with flow cytometry. The percentage of internalized bacteria was variable. SH1 again showed a lower invasion rate compared to SH2, which demonstrated a higher invasion rate (Figure 4**)**. Again the clinical isolates had superior cellular invasion abilities compared to the control SH strain. Summary of the PHSF FITC^+^ cells that are infected with different isolates is shown in Table 3.

***S. haemolyticus* efficiently invade the PHSF cells**

We next aimed to quantify the CFU that invaded the PHFS cells. To do that, we challenged the PHSF cells with the *S. haemolyticus* strains for 3 h and then treated the cells with lysostaphin to lyse the extracellular or adherent staphylococci. Most *S. haemolyticus* strains effectively invaded the PHSF cells and achieved high intracellular levels. Of note, SH1 which was negative for both *fnbA* and *fnbB,* showed a low invasion capacity (Figure 5). SH2, SH7, SH9 and SH10 carried both the *fnbA* and *fnbB* genes and showed a higher level of invasion compared to other isolates. Mean percentage of internalized bacteria= 1.56±0.06 and 2±0.36 in case of cells infected with bacteria that carry *fnbB* only or infected with bacteria that carry both *fnbA* and *fnbB* genes, respectively (p value = 0.027 calculated with unpaired *t-*test). The *fnBP* genes are probably not the only determinants of the invasion capabilities of the *S. haemolyticus* to the PHSF cells because SH1 strain, which did not carry the *fnbA* or *fnbB* gene was also able to invade and enter the cells although to a lower level. Of note, some strains carried a low number of virulence genes but were still able to invade the skin fibroblasts to remarkable levels (e.g SH4, SH5, SH6 and SH8) (Table 3).

***S. haemolyticus* and their cell-free supernatants affect the proliferation rate of the PHSF cells**

Since *S. haemolyticus* was able to invade the PHSF cells, we wondered whether *S. haemolyticus* can affect the viability of the infected cells. Therefore, the MTT assay was performed to study the effect of *S. haemolyticus* or its products on the viability of PHSF cells. *S. aureus* and control *S. haemolyticus* were used for comparison purposes. The capacity of bacterial suspension, cell-free supernatants or heated bacteria to impair the viability of PHSF cells was determined at 6, 18, and 24 h post-infection. Our results showed that bacterial suspensions and cell-free supernatants of the clinical *S. haemolyticus* strains exhibited high cytotoxicity compared to the control *S. haemolyticus* strain. However, this cytotoxic effect was inhibited when cells were heated at 65 ˚C for 30 min.

The decrease in PHSF viability occurred in a time-dependent manner. At approximately only 6 h after adding the bacteria or the filtered supernatants, the percentage of viable cells was markedly reduced, and reached a dramatic level after 18 h (Figure 6). Generally, the effects of the bacterial suspensions and the cell-free supernatants of *S. haemolyticus* and *S. aureus* were similar. The mean percentages of viable PHSF cells infected with whole *S. haemolyticus* suspensions were 54.2%±5.4, 19.6%±2.1 and 21.7%±5.2 at 6, 18 and 24 h post-infection, respectively. Similarly, the mean percentage of viable PHSF cells infected with whole *S. aureus* isolates were 54.3%±12.7, 13.5%±1.2 and 7.0%±0.7 after 6, 18 and 24 h of infection. When cells were treated with *S. haemolyticus* supernatants, 86.2%±3.4, 14.0%±1.4 and 13.9%±1.7 were viable after 6, 18 and 24 h of infection, respectively. The values were comparable to those obtained when cells were treated with *S. aureus* supernatants (77.9%±4.3, 16.9%±2.8 and 14.1%±0.3 after 6, 18 and 24 h of infection, respectively).

***S. haemolyticus* and their cell-free supernatants induce apoptosis in the PHSF cells**

We determined whether *S. haemolyticus* can induce apoptosis in the PHSF cells. Flow cytometry using FITC-conjugated annexin V and PI staining revealed that PHSF cells exposed to the clinical strains of *S. haemolyticus* or *S. haemolyticus* supernatants underwent rapid apoptosis (Figure 7). The control *S. haemolyticus* (ATCC 29970) was unable to induce significant cytotoxicity in the PHSF cells. Similar results were observed when fibroblasts were challenged with the bacterial supernatant or when heat treated.

The mean percentage of apoptotic PHSF cells challenged with *S. haemolyticus* was highly similar to that obtained when cells were challenged with *S. aureus* (40.9%±15 and 42.3%±5.3, respectively, p-value > 0.05). However, more apoptotic cells were detected when PHSF cells were treated with SA supernatants (62.7% ± 3.75) than with SH supernatant (35.9% ± 11.2) (p value = 0.02). On the other hand, heat-treated SH and SA did not induce significant apoptosis.

**Analysis of cytokine expression**

The expression of different cytokines by PBMCs was quantified after 6 and 24 h of co-culturing with *S. haemolyticus* or *S. aureus*. Both bacteria induced the expression of all tested cytokines after 6 h, which generally increased after 24 h (Figure 8). After 6 h of co-culture, *S. haemolyticus* induced higher levels of the inflammatory cytokines IL1β, IL4 and IFNγ compared to *S. aureus.* However*, S. haemolyticus* induced lower levels of the pro-inflammatory cytokines IL17 and TNFα. Also, higher levels of the anti-inflammatory cytokines IL10 and TGFβ were observed when cells were co-cultured with *S. haemolyticus.* While, after 24 h, *S. haemolyticus* induced lower levels of the pro-inflammatory cytokines IL1β, IL4, IL17, IFNγ, TNFα and the anti-inflammatory TGFβ compared to *S. aureus.* However, *S. haemolyticus* induced higher levels of the anti-inflammatory cytokines IL10.

**Discussion**

Our study described for the first time the pathogenesis of S. haemolyticus isolated from patients with DFUs on PHSF cells. Staphylococci, including S. haemolyticus, are widely distributed in hospital environment and are important causative agents of DFUs [[23](#_ENREF_23), [24](#_ENREF_24)]. We employed an explant culture method for the isolation of the PHSF cells from human skin, which is a simple*,* reliable and inexpensive method for isolation of skin fibroblasts [[25](#_ENREF_25)]. Keratinocytes did not attach to tissue culture plates and were removed by washing with PBS because keratinocytes need additional nutritional supplements and growth factors that were not included in our media [[26](#_ENREF_26)].

**Detection of the virulence-related genes**

The pathogenicity of *S. haemolyticus* isolates was directly associated with a broad spectrum of virulence factors which play a central role in the pathogenesis of staphylococcal infections. DFU is one of the leading causes of severe complications. These ulcers are caused by microorganisms that may carry different virulence factors. In this study, we analyzed a pool of *S. haemolyticus* isolates for the presence of genes encoding the enterotoxin, hemolysin, fibronectin-binding protein, and exfoliative toxins genes by PCR using pairs of specific primers.

Most *S. haemolyticus* isolates contained at least one type of the enterotoxin genes, and one isolate contained four different types. Staphylococcal enterotoxins (SE) constitute a family of nine major serological types of heat-stable enterotoxins that are biologically and structurally related. The most prevalent enterotoxin types were seg, sea and sec genes. Importantly, [Skov, Olsen [27]](#_ENREF_27) reported that the application of the staphylococcal enterotoxin seb on human skin led to the induction of dermatitis, which may contribute to the damage induced by the infection of the diabetic foot ulcers. The ingestion of these toxins results in gastrointestinal manifestations such as nausea, vomiting, diarrhea, and abdominal pain. SEs are the leading cause of bacterial food poisoning in human beings and have been described as the cause of many outbreaks of foodborne diseases [[28](#_ENREF_28)]. Moreover, these toxins are powerful superantigens that stimulate non-specific T-cell proliferation and may lead to toxic shock syndrome. Secreted enterotoxins are the major weapons that kill host cells and cause diseases by inducing different types of cell death, particularly apoptosis and necrosis [[29](#_ENREF_29)].

**Detection of the fibronectin-binding protein (FnBP) genes and adhesion assay**

*Staphylococci* can express a variety of virulence factors, including surface proteins such as the FnBPs, which include *fnbA* and *fnbB*. These adhesive proteins play a crucial role in bacterial binding and adherence to the extracellular matrix and, accordingly, host cell invasion and bacterial internalization into cells [[30-32](#_ENREF_30)]. Since keratinocytes and skin fibroblasts produce fibronectin and fibrinogen for the promotion of wound healing and tissue repair, these proteins will serve as a target or bacterial FnBPs [[33](#_ENREF_33)]. [Pereira, Teixeira [34]](#_ENREF_34) demonstrated that in *S. aureus*, increased virulence has been associated with its adhesive properties. Moreover, the fnbB genes were also involved in intercellular accumulation and development of biofilms [[35](#_ENREF_35)]. Microbial infections resulting from bacterial adhesion to biomaterial surfaces have been observed on almost all medical devices [[36](#_ENREF_36)].

We found that most *S. haemolyticus* isolates possessed either fnbA or fnbB or both*.* fnbA and fnbB genes were absent in SH1. Interestingly, this strain had low ability of invasion into the PHSF cells, pointing to the vital role of fnbA and fnbB genes in adhesion to host cells. Similar to our observation, [Tuchscherr, Korpos [5]](#_ENREF_5) showed that the lack of human umbilical vein endothelial cells (HUVECs) invasiveness in *S. aureus* isolates was due to defects in FnBPs. The *fnBP* genes are commonly detected in *S. aureus* and different researchers reported variable percentages of the fnb*A* and fnb*B* genes. [Tristan, Ying [37]](#_ENREF_37) demonstrated that 43% of *S. aureus* were positive for *fnbB* and 28% were positive for *fnbA.* In 72 Tunisian [MRSA](https://www.sciencedirect.com/topics/medicine-and-dentistry/methicillin-resistant-staphylococcus-aureus) isolates, fnb*A* gene was detected in 12 strains, fnb*B* gene in 2 strains, and both fnb*A* and fnb*B* genes in 2 other strains [[38](#_ENREF_38)]. Also, [Mirzaee, Najar-Peerayeh [39]](#_ENREF_39) showed that the prevalence of *fnbA* and *fnbB* in MRSA strains was 82.2% and 46.7%, respectively.

Although fibronectin adhesion is a major explanation for *S. aureus* virulence, CoNS internalization is a more controversial issue [[40](#_ENREF_40)]. Interestingly, [Switalski, Ryden [41]](#_ENREF_41) showed that *S. haemolyticus* isolates were able to bind fibronectin. Moreover, *S. haemolyticus* could bind collagen and vitronectin in a time-dependent manner [[42](#_ENREF_42)]. A recent study has examined the binding of *S. haemolyticus* to human keratinocytes and reported a better adherence of clinical *S. haemolyticus* compared to commensal strains*.* Surface shaving analysis identified 65 surface proteins, including 3 adhesins, extracellular matrix binding protein (Embp), Mannosylglucosyl-3-phosphoglycerate phosphatase (SasH-like) and others. Upon colonization, an increase in *SceD* and the autolysin *Atl* genes was observed [[43](#_ENREF_43)]. In fact, no previous reports have clearly detected FnBP proteins in CoNS [[44](#_ENREF_44)]*.* Therefore, our results propose a new mechanism for adhesion and internalization of *S. haemolyticus.* However, more experiments are required to test the expression and upregulation of these genes upon infection of the cells. S. epidermidis has been shown to bind fibronectin by the extracellular matrix binding protein (Embp), which facilities adhesion to fibronectin and formation of biofilm layers. One of the proposed explanations is that *S. epidermidis* enters the cells through a tripartite Embp-Fn-α5β1 system which is analogous to the FnBP-Fn-α5β1 integrin in *S. aureus* [[45](#_ENREF_45)]. Indeed, [Campoccia, Testoni [46]](#_ENREF_46) has found that *S. epidermidis* internalization was 100 times lower than *S. aureus* internalization.

**Invasive of the PHSF cells by *S. haemolyticus***

The ability of *S. aureus* to be internalized by host cells is considered one of the most critical pathogenicity factors in persisting and relapsing infections [[44](#_ENREF_44)]. Trends of invasion and intracellular survival were similar in flow cytometry and lysostaphin protection experiments. All *S. haemolyticus* isolates could invade the PHSF cells to varying degrees. It seems that fibronectin-binding proteins of *S. haemolyticus* are essential in the process of internalization by PHSF cells since SH1, which was deficient in both genes, produced the least ability of adhesion and invasion. However, the presence of at least one of the known FnBP was significantly associated with marked adhesion and invasion of PHSF cells. Strains that carry both genes invaded the PHSF cells at a significantly higher level than the other strains. However, more studies are required to test and to quantitatively analyze the expression of the *FnBP* upon infection of the PHSF cells by *S. haemolyticus*.

**Cytotoxicity and apoptosis**

The effect of *S. haemolyticus* on the viability of PHSF cells was tested by observing two outcomes; the reduction of cell viability and proliferation measured by MTT assay and induction of apoptosis, which was measured by Annexin V/PI staining using flow cytometry. We tested whether the cytotoxic effects were caused by secreted factors or caused only by direct cell invasion ~~we tested the impact of cell-free filtered bacterial supernatants on the cell viability.~~ The bacterial cells caused cytotoxic effects similar to those obtained by the cell-free supernatants. This means that viable metabolically active bacteria were not necessary for the cell-killing ability of the tested isolates and that the bacterial products induced cytotoxic effects, which were comparable to that induced by cellular invasion. The effects on cell viability and apoptosis induced by *S. haemolyticus* were comparable to the levels induced by *S. aureus* strains. However, the induction of cytotoxicity was lost when cells were incubated with heat-treated bacteria (65 °C for 30 min), indicating the heat-labile properties of the toxic bacterial products.

The cytotoxic effect induced by bacteria is highly dependent on the virulence of the tested microorganism and the cell line [[47](#_ENREF_47)]. This study was the first report that described the cytotoxicity of *S. haemolyticus* bacteria on PHSF cells. In our study, *S. haemolyticus* isolates exhibited different levels of cytotoxic activity ranging from 11.2% to 80.4%. The cytotoxic activity of whole *S. haemolyticus* and cell-free supernatants were similar to that induced by whole *S. aureus* and their supernatants. *S. haemolyticus* and the filtered bacterial supernatant remarkably decreased proliferation and increased apoptosis of the PHSF cells, with different degrees. In another study, *S. haemolyticus* was able to induce cytotoxicity of the HEp-2 cells (human epidermoid carcinoma cells from the larynx) that ranged from 13.8 to 81.5 %. In the same study, preheating of the cell-free supernatants at 56 ˚C reduced the cytotoxic activity from 7.1% ±1.7 % to 19.6% ±2.9 % [[18](#_ENREF_18)]. Moreover, [Krzyminska, Szczuka [48]](#_ENREF_48) showed that all strains of S. haemolyticus exhibited cytotoxic effects on murine macrophage cell line J774, which were evident by the detachment of the cells from the surface of the wells. They also reported that three strains (10 %) showed low cytotoxic activity.

In contrast to our results, [Johansson, Rautelin [49]](#_ENREF_49) demonstrated that *S. aureus* isolates were not cytotoxic to HeLa or HT29 cells. Also, [El-Housseiny, Aboulwafa [50]](#_ENREF_50) showed that the cell-free supernatants of the four *E. coli* isolates caused nearly no cytotoxicity after 3 h of Vero cell infection. On the other hand, the washed bacterial cells caused high cytotoxic effects similar to those obtained by the whole culture. In another study, the filtered supernatant from S. aureus failed to induce apoptosis when applied to endothelial cell monolayers, thereby excluding any effect from the preformed α-toxin or other soluble bacterial factors [[51](#_ENREF_51)]. Also, [van Kruchten, Wilden [52]](#_ENREF_52) showed that human alveolar basal epithelial cell line (A549), bronchial epithelial cell line (Calu-3), primary human umbilical vein endothelial cells (HUVECs) and human bronchial epithelial cells (HBEpCs) infected with *S. aureus* alone displayed no apoptosis. Heat treating of *S. haemolyticus* markedly interfered with the bacterial cytotoxic potential on the PHSF cells. In line with our results, [Kahl, Goulian [53]](#_ENREF_53) showed that apoptosis induced in epithelial cells by infection with heat-killed S. aureus did not manifest any DNA fragmentation. Contrary to our results, [Ocana, Asensi [54]](#_ENREF_54) showed that the induction of apoptosis in neutrophil incubated with bacteria did not require live bacteria since organisms killed by heat treatment induced the same apoptotic effect.

**Detection of hemolysin and *S. haemolyticus* hemolytic activity**

Our results showed that different levels of hemolytic activities were detected in all *Staphylococcus* isolates. It is generally considered that *S. aureus* produces four types of hemolysins (alpha, beta, gamma, and delta), which have hemolytic and cytotoxic effects. In particular, the *α-hemolysin* (*hla*) gene causes significant hemolysis [[55](#_ENREF_55)]. Moreover, *hla* of *S. aureus* was shown to induce apoptosis in peripheral T lymphocytes [[56](#_ENREF_56)]. The toxin also disrupts the tissue barrier at host interfaces lined by epithelial or endothelial cells [[57](#_ENREF_57)]. [Seidl, Leemann [58]](#_ENREF_58) showed that *S. aureus* isolates with α-toxin production induced significantly more endothelial damage compared to isolates without α-toxin production. Our results are in agreement with others who reported that *hla* is the most common *hemolysin* gene in *Staphylococci*. [Moraveji, Tabatabaei [59]](#_ENREF_59) reported that *hla* is more frequently expressed by *S. haemolyticus* isolated from ulcers of diabetic patients. [Pinheiro, Brito [60]](#_ENREF_60) showed that the *hla* gene was present in 91.7% of *S. haemolyticus* isolates. Also, [Alfatemi, Motamedifar [61]](#_ENREF_61) showed that 93.15% *S. aureus* isolates were positive for the *hla* gene.

**Impact of *S. haemolytics* infection on cytokine expression**

In order to improve our knowledge of the interaction between the immune system and *S. haemolyticus* during the infection, we investigated the expression of IL1β, IL4, IL17, IFNγ, TNFα, TGFβ and IL10 by PBMCs infected with *S*. *haemolyticus*. *S. haemolyticus* and *S. aureus* induced human PBMCs to produce different cytokines at different relative levels after 6 and 24 h of infection. We observed a marked early inflammatory response, which is consistent with other reports that described a similar production of high levels of inflammatory mediators (IL1β, IL6, and TNFα) after *S. aureus* peritoneal infection [[62](#_ENREF_62)]. This could be explained by the expression of different types of Toll-like receptors on PBMCs, which interacts with the bacteria and its products, leading to an increase in inflammatory cytokine release [[63](#_ENREF_63)]. Similar to our observation, stimulation of the keratinocytes with S. epidermidis evoked the gene expression and release of the powerful pro-inflammatory IL1β beta [[64](#_ENREF_64)]. IL17 plays a key role in the defense of the host against different pathogens, including bacteria and viruses [[65](#_ENREF_65)]. IL17 also produces a synergistic effect with other cytokines, such as IL1, IL6 and TNFα, to enhance the ability of the tissue infiltrating neutrophils to clear the extracellular pathogens [[66](#_ENREF_66)]. [Islander, Andersson [67]](#_ENREF_67) reported that superantigens produced by *S. aureus* were efficient in stimulating IL17 release. According to our results, IL4 expression was greater than IL1. [Giese, Sumner [68]](#_ENREF_68) reported that IL4 might induce inhibitory effects on IL1β production, resulting in a reduced level.

Staphylococci also induced a marked increase in the expression of the down regulatory IL10 cytokine, particularly after 24 h, which attenuated the inflammatory response and may aid in improving the skin swelling, erythema and inflammation [[69](#_ENREF_69)]. The peptidoglycan in the cell wall of Staphylococci induces the release of known immunosuppressive mediators such as IL10, PGE_2_, and TGFβ [[1](#_ENREF_1)]. IL10 may play a protective role by inhibiting Th1 responses and blocking the expression of the pro-inflammatory cytokines [[70](#_ENREF_70)]. Since IL10 is known to inhibit the synthesis of IFNγ as well as IL1, IL6, IL8 and TNFα. IL10 may dampen the inflammation and cytotoxic effect of these cytokines, which prevents chronic inflammation and host morbidity. On the other hand, the attenuation of the immune responses may lead to an increase in bacterial load [[71](#_ENREF_71), [72](#_ENREF_72)]. We noticed that TNF levels did not increase further after 24 h of infection compared to their levels after 6 h. It is possible that the marked increase in IL10 levels hindered the further increase in TNF expression levels.

We concluded that *S. haemolyticus* carries an inclusive set of genes that code for different virulence factors such as toxins, enzymes and adhesion proteins. Our results contribute to a better understanding of the pathomechanisms of *S. haemolyticus* infections and fill a gap in the literature regarding the CoNS. Based on our observations, microbiology laboratories should consider *S. haemolyticus* as a critical opportunistic pathogen and infections caused by this organism, particularly in critically ill patients, should be seriously managed. Additional experiments are planned to study the expression of these genes upon infection of the PHSF cells and the role of these genes in the invasion, intracellular survival and establishment of infection in the host cells. These factors may contribute to the bacteria’s ability to spread through tissues and the pathological damage associated with DFU. Among the limitations of this study is the low number of tested isolates and that the all tested bacteria were obtained from only one site of infection. However, in future studies we will test the pathogenesis of more clinical strains that are collected from different sites of infection

**Acknowledgment**: Authors acknowledge the Medical Research Center at the Faculty of Medicine, Assiut University for providing the necessary research equipment.

**Declaration of interest statement:** authors have no conflict of interest.

**References**

1. Czekaj, T., M. Ciszewski, and E.M. Szewczyk, *Staphylococcus haemolyticus - an emerging threat in the twilight of the antibiotics age.* Microbiology, 2015. 161(11): p. 2061-8.

2. Silva, P.V., R.S. Cruz, L.S. Keim, et al., *The antimicrobial susceptibility, biofilm formation and genotypic profiles of Staphylococcus haemolyticus from bloodstream infections.* Mem Inst Oswaldo Cruz, 2013. 108(6): p. 812-3.

3. do Carmo Ferreira, N., R.P. Schuenck, K.R. dos Santos, et al., *Diversity of plasmids and transmission of high-level mupirocin mupA resistance gene in Staphylococcus haemolyticus.* FEMS Immunol Med Microbiol, 2011. 61(2): p. 147-52.

4. Schuenck, R.P., E.M. Pereira, N.L. Iorio, et al., *Multiplex PCR assay to identify methicillin-resistant Staphylococcus haemolyticus.* FEMS Immunol Med Microbiol, 2008. 52(3): p. 431-5.

5. Tuchscherr, L., E. Korpos, H. van de Vyver, et al., *Staphylococcus aureus requires less virulence to establish an infection in diabetic hosts.* Int J Med Microbiol, 2018. 308(7): p. 761-769.

6. Citron, D.M., E.J. Goldstein, C.V. Merriam, et al., *Bacteriology of moderate-to-severe diabetic foot infections and in vitro activity of antimicrobial agents.* J Clin Microbiol, 2007. 45(9): p. 2819-28.

7. Rossi, C.C., J.F. Santos-Gandelman, E.M. Barros, et al., *Staphylococcus haemolyticus as a potential producer of biosurfactants with antimicrobial, anti-adhesive and synergistic properties.* Lett Appl Microbiol, 2016. 63(3): p. 215-21.

8. Takeuchi, F., S. Watanabe, T. Baba, et al., *Whole-genome sequencing of staphylococcus haemolyticus uncovers the extreme plasticity of its genome and the evolution of human-colonizing staphylococcal species.* J Bacteriol, 2005. 187(21): p. 7292-308.

9. Ahmed, A., L. Satti, G. Zaman, et al., *Catheter related recurrent blood stream infection caused by linezolid-resistant, methicillin resistant Staphylococcus haemolyticus; an emerging super bug.* J Pak Med Assoc, 2019. 69(2): p. 261-263.

10. Sader, H.S., R.N. Jones, A.C. Gales, et al., *SENTRY antimicrobial surveillance program report: Latin American and Brazilian results for 1997 through 2001.* Braz J Infect Dis, 2004. 8(1): p. 25-79.

11. Anthonisen, I.L., M. Sunde, T.M. Steinum, et al., *Organization of the antiseptic resistance gene qacA and Tn552-related beta-lactamase genes in multidrug- resistant Staphylococcus haemolyticus strains of animal and human origins.* Antimicrob Agents Chemother, 2002. 46(11): p. 3606-12.

12. Barros, E.M., M. Lemos, T. Souto-Padron, et al., *Phenotypic and genotypic characterization of biofilm formation in Staphylococcus haemolyticus.* Curr Microbiol, 2015. 70(6): p. 829-34.

13. Keira, S.M., L.M. Ferreira, A. Gragnani, et al., *Experimental model for fibroblast culture.* Acta Cirurgica Brasileira, 2004. 19(suppl 1): p. 11-16.

14. Vangipuram, M., D. Ting, S. Kim, et al., *Skin punch biopsy explant culture for derivation of primary human fibroblasts.* J Vis Exp, 2013(77): p. e3779.

15. Pinheiro, L., C.I. Brito, A. Oliveira, et al., *Staphylococcus epidermidis and Staphylococcus haemolyticus: detection of biofilm genes and biofilm formation in blood culture isolates from patients in a Brazilian teaching hospital.* Diagn Microbiol Infect Dis, 2016. 86(1): p. 11-4.

16. Cremet, L., A. Broquet, B. Brulin, et al., *Pathogenic potential of Escherichia coli clinical strains from orthopedic implant infections towards human osteoblastic cells.* Pathog Dis, 2015. 73(8): p. ftv065.

17. Guglielmetti, S., V. Taverniti, M. Minuzzo, et al., *Oral bacteria as potential probiotics for the pharyngeal mucosa.* Appl Environ Microbiol, 2010. 76(12): p. 3948-58.

18. Krzyminska, S., E. Szczuka, K. Dudzinska, et al., *Virulence and the presence of aminoglycoside resistance genes of Staphylococcus haemolyticus strains isolated from clinical specimens.* Antonie Van Leeuwenhoek, 2015. 107(4): p. 857-68.

19. Schrand, A.M., J.J. Schlager, L. Dai, et al., *Preparation of cells for assessing ultrastructural localization of nanoparticles with transmission electron microscopy.* Nature protocols, 2010. 5(4): p. 744-757.

20. Juuti, K.M., B. Sinha, C. Werbick, et al., *Reduced adherence and host cell invasion by methicillin-resistant Staphylococcus aureus expressing the surface protein Pls.* J Infect Dis, 2004. 189(9): p. 1574-84.

21. Cheung, A.L. and K.W. Bayles, *Tissue culture assays used to analyze invasion by Staphylococcus aureus.* Curr Protoc Microbiol, 2007. Chapter 9: p. Unit 9C 4.

22. Saliba, A.M., A. Filloux, G. Ball, et al., *Type III secretion-mediated killing of endothelial cells by Pseudomonas aeruginosa.* Microb Pathog, 2002. 33(4): p. 153-66.

23. Sievert, D.M., P. Ricks, J.R. Edwards, et al., *Antimicrobial-resistant pathogens associated with healthcare-associated infections: summary of data reported to the National Healthcare Safety Network at the Centers for Disease Control and Prevention, 2009-2010.* Infect Control Hosp Epidemiol, 2013. 34(1): p. 1-14.

24. El-Mokhtar, M.A. and H.F. Hetta, *Ambulance vehicles as a source of multidrug-resistant infections: a multicenter study in Assiut City, Egypt.* Infect Drug Resist, 2018. 11: p. 587-594.

25. Nejaddehbashi, F., V. Bayati, L. Mashali, et al., *Isolating human dermal fibroblasts using serial explant culture.* Stem cell investigation, 2019. 6: p. 23-23.

26. Marcelo, C.L., A. Peramo, A. Ambati, et al., *Characterization of a unique technique for culturing primary adult human epithelial progenitor/"stem cells".* BMC Dermatol, 2012. 12: p. 8.

27. Skov, L., J.V. Olsen, R. Giorno, et al., *Application of Staphylococcal enterotoxin B on normal and atopic skin induces up-regulation of T cells by a superantigen-mediated mechanism.* J Allergy Clin Immunol, 2000. 105(4): p. 820-6.

28. Basso, A.P., P.D. Martins, G. Nachtigall, et al., *Antibiotic resistance and enterotoxin genes in Staphylococcus sp. isolates from polluted water in Southern Brazil.* An Acad Bras Cienc, 2014. 86(4): p. 1813-20.

29. Lin, C.F., C.L. Chen, W.C. Huang, et al., *Different types of cell death induced by enterotoxins.* Toxins (Basel), 2010. 2(8): p. 2158-76.

30. Foster, T.J. and M. Hook, *Surface protein adhesins of Staphylococcus aureus.* Trends Microbiol, 1998. 6(12): p. 484-8.

31. Musyoki, A.M., Z. Shi, C. Xuan, et al., *Structural and functional analysis of an anchorless fibronectin-binding protein FBPS from Gram-positive bacterium Streptococcus suis.* Proc Natl Acad Sci U S A, 2016. 113(48): p. 13869-13874.

32. McElroy, M.C., D.J. Cain, C. Tyrrell, et al., *Increased virulence of a fibronectin-binding protein mutant of Staphylococcus aureus in a rat model of pneumonia.* Infect Immun, 2002. 70(7): p. 3865-73.

33. Planet, P.J., S.J. LaRussa, A. Dana, et al., *Emergence of the epidemic methicillin-resistant Staphylococcus aureus strain USA300 coincides with horizontal transfer of the arginine catabolic mobile element and speG-mediated adaptations for survival on skin.* mBio, 2013. 4(6): p. e00889-13.

34. Pereira, E.M., C.A.A. Teixeira, A.L.M. Alvarenga, et al., *A Brazilian lineage of Staphylococcus lugdunensis presenting rough colony morphology may adhere to and invade lung epithelial cells.* J Med Microbiol, 2012. 61(Pt 4): p. 463-469.

35. Szczuka, E., K. Urbanska, M. Pietryka, et al., *Biofilm density and detection of biofilm-producing genes in methicillin-resistant Staphylococcus aureus strains.* Folia Microbiol (Praha), 2013. 58(1): p. 47-52.

36. Rodrigues, L.R., *Inhibition of bacterial adhesion on medical devices.* Adv Exp Med Biol, 2011. 715: p. 351-67.

37. Tristan, A., L. Ying, M. Bes, et al., *Use of multiplex PCR to identify Staphylococcus aureus adhesins involved in human hematogenous infections.* J Clin Microbiol, 2003. 41(9): p. 4465-7.

38. Ben Nejma, M., M. Mastouri, S. Frih, et al., *Molecular characterization of methicillin-resistant Staphylococcus aureus isolated in Tunisia.* Diagn Microbiol Infect Dis, 2006. 55(1): p. 21-6.

39. Mirzaee, M., S. Najar-Peerayeh, and M. Behmanesh, *Prevalence of fibronectin-binding protein (FnbA and FnbB) genes among clinical isolates of methicillin resistant Staphylococcus aureus.* Molecular Genetics, Microbiology and Virology, 2015. 30(4): p. 221-224.

40. Josse, J., F. Laurent, and A. Diot, *Staphylococcal Adhesion and Host Cell Invasion: Fibronectin-Binding and Other Mechanisms.* Front Microbiol, 2017. 8: p. 2433.

41. Switalski, L.M., C. Ryden, K. Rubin, et al., *Binding of fibronectin to Staphylococcus strains.* Infect Immun, 1983. 42(2): p. 628-33.

42. Paulsson, M. and T. Wadstrom, *Vitronectin and type-I collagen binding by Staphylococcus aureus and coagulase-negative staphylococci.* FEMS Microbiol Immunol, 1990. 2(1): p. 55-62.

43. Wolden, R., M. Pain, R. Karlsson, et al., *Identification of surface proteins in a clinical Staphylococcus haemolyticus isolate by bacterial surface shaving.* BMC Microbiol, 2020. 20(1): p. 80.

44. Hirschhausen, N., T. Schlesier, M.A. Schmidt, et al., *A novel staphylococcal internalization mechanism involves the major autolysin Atl and heat shock cognate protein Hsc70 as host cell receptor.* Cell Microbiol, 2010. 12(12): p. 1746-64.

45. Khalil, H., R.J. Williams, G. Stenbeck, et al., *Invasion of bone cells by Staphylococcus epidermidis.* Microbes Infect, 2007. 9(4): p. 460-5.

46. Campoccia, D., F. Testoni, S. Ravaioli, et al., *Orthopedic implant infections: Incompetence of Staphylococcus epidermidis, Staphylococcus lugdunensis, and Enterococcus faecalis to invade osteoblasts.* J Biomed Mater Res A, 2016. 104(3): p. 788-801.

47. Strobel, M., H. Pfortner, L. Tuchscherr, et al., *Post-invasion events after infection with Staphylococcus aureus are strongly dependent on both the host cell type and the infecting S. aureus strain.* Clin Microbiol Infect, 2016. 22(9): p. 799-809.

48. Krzyminska, S., E. Szczuka, and A. Kaznowski, *Staphylococcus haemolyticus strains target mitochondria and induce caspase-dependent apoptosis of macrophages.* Antonie Van Leeuwenhoek, 2012. 102(4): p. 611-20.

49. Johansson, C., H. Rautelin, and R. Kaden, *Staphylococcus argenteus and Staphylococcus schweitzeri are cytotoxic to human cells in vitro due to high expression of alpha-hemolysin Hla.* Virulence, 2019. 10(1): p. 502-510.

50. El-Housseiny, G.S., M.M. Aboulwafa, and N.A. Hassouna, *Cytotoxic activities of some Escherichia coli isolates: possible mechanisms and approaches for inhibition.* Journal of American Science, 2010. 6(10): p. 269-283.

51. Menzies, B.E. and I. Kourteva, *Staphylococcus aureus alpha-toxin induces apoptosis in endothelial cells.* FEMS immunology and medical microbiology, 2000. 29(1): p. 39-45.

52. van Kruchten, A., J.J. Wilden, S. Niemann, et al., *Staphylococcus aureus triggers a shift from influenza virus-induced apoptosis to necrotic cell death.* FASEB J, 2018. 32(5): p. 2779-2793.

53. Kahl, B.C., M. Goulian, W. van Wamel, et al., *Staphylococcus aureus RN6390 Replicates and Induces Apoptosis in a Pulmonary Epithelial Cell Line.* Infection and Immunity, 2000. 68(9): p. 5385-5392.

54. Ocana, M.G., V. Asensi, A.H. Montes, et al., *Autoregulation mechanism of human neutrophil apoptosis during bacterial infection.* Mol Immunol, 2008. 45(7): p. 2087-96.

55. Lee, J.H., Y.G. Kim, S. Yong Ryu, et al., *Calcium-chelating alizarin and other anthraquinones inhibit biofilm formation and the hemolytic activity of Staphylococcus aureus.* Sci Rep, 2016. 6(1): p. 19267.

56. Breuer, K., M. Wittmann, K. Kempe, et al., *Alpha-toxin is produced by skin colonizing Staphylococcus aureus and induces a T helper type 1 response in atopic dermatitis.* Clin Exp Allergy, 2005. 35(8): p. 1088-95.

57. Berube, B.J. and J. Bubeck Wardenburg, *Staphylococcus aureus alpha-toxin: nearly a century of intrigue.* Toxins (Basel), 2013. 5(6): p. 1140-66.

58. Seidl, K., M. Leemann, M. Palheiros Marques, et al., *High level methicillin resistance correlates with reduced Staphylococcus aureus endothelial cell damage.* International journal of medical microbiology : IJMM, 2017. 307(1): p. 11-20.

59. Moraveji, Z., M. Tabatabaei, H. Shirzad Aski, et al., *Characterization of hemolysins of Staphylococcus strains isolated from human and bovine, southern Iran.* Iran J Vet Res, 2014. 15(4): p. 326-30.

60. Pinheiro, L., C.I. Brito, A. de Oliveira, et al., *Staphylococcus epidermidis and Staphylococcus haemolyticus: Molecular Detection of Cytotoxin and Enterotoxin Genes.* Toxins (Basel), 2015. 7(9): p. 3688-99.

61. Alfatemi, S.M.H., M. Motamedifar, N. Hadi, et al., *Analysis of Virulence Genes Among Methicillin Resistant Staphylococcus aureus (MRSA) Strains.* Jundishapur Journal of Microbiology, 2014. 7(6).

62. de Souza, P.R.K., S.S. Ferreira, F.P.B. Nunes, et al., *Cytokine and Adhesion Molecule Expression Induced by Different Strains of Staphylococcus aureus in Type 1 Diabetic Rats: Role of Insulin.* Front Immunol, 2018. 9: p. 3165.

63. Musa, H.H., S.L. Wu, C.H. Zhu, et al., *Immune response of peripheral blood mononuclear cells to avian pathogenic Escherichia coli.* Annals of Microbiology, 2009. 59(3): p. 587-592.

64. Simanski, M., A.-S. Erkens, F. Rademacher, et al., *Staphylococcus epidermidis-induced Interleukin-1 Beta and Human Beta-defensin-2 Expression in Human Keratinocytes is Regulated by the Host Molecule A20 (TNFAIP3).* Acta dermato-venereologica, 2019. 99(2): p. 181-187.

65. Li, Y., C. Wei, H. Xu, et al., *The Immunoregulation of Th17 in Host against Intracellular Bacterial Infection.* Mediators Inflamm, 2018. 2018: p. 6587296.

66. Valeri, M. and M. Raffatellu, *Cytokines IL-17 and IL-22 in the host response to infection.* Pathog Dis, 2016. 74(9).

67. Islander, U., A. Andersson, E. Lindberg, et al., *Superantigenic Staphylococcus aureus stimulates production of interleukin-17 from memory but not naive T cells.* Infect Immun, 2010. 78(1): p. 381-6.

68. Giese, M.J., H.L. Sumner, J.A. Berliner, et al., *Cytokine expression in a rat model of Staphylococcus aureus endophthalmitis.* Investigative ophthalmology & visual science, 1998. 39(13): p. 2785-2790.

69. Hendricks, A.J., B.W. Mills, and V.Y. Shi, *Skin bacterial transplant in atopic dermatitis: Knowns, unknowns and emerging trends.* J Dermatol Sci, 2019. 95(2): p. 56-61.

70. Van Belleghem, J.D., F. Clement, M. Merabishvili, et al., *Pro- and anti-inflammatory responses of peripheral blood mononuclear cells induced by Staphylococcus aureus and Pseudomonas aeruginosa phages.* Sci Rep, 2017. 7(1): p. 8004.

71. Leech, J.M., K.A. Lacey, M.E. Mulcahy, et al., *IL-10 Plays Opposing Roles during Staphylococcus aureus Systemic and Localized Infections.* J Immunol, 2017. 198(6): p. 2352-2365.

72. Elgendy, S.G., M.R. Abdel Hameed, and M.A. El-Mokhtar, *Tigecycline resistance among Klebsiella pneumoniae isolated from febrile neutropenic patients.* J Med Microbiol, 2018. 67(7): p. 972-975.

73. Seng, R., T. Kitti, R. Thummeepak, et al., *Biofilm formation of methicillin-resistant coagulase negative staphylococci (MR-CoNS) isolated from community and hospital environments.* PLoS One, 2017. 12(8): p. e0184172.

74. Kalinka, J., M. Hachmeister, J. Geraci, et al., *Staphylococcus aureus isolates from chronic osteomyelitis are characterized by high host cell invasion and intracellular adaptation, but still induce inflammation.* Int J Med Microbiol, 2014. 304(8): p. 1038-49.

75. Jarraud, S., C. Mougel, J. Thioulouse, et al., *Relationships between Staphylococcus aureus genetic background, virulence factors, agr groups (Alleles), and human disease.* Infection and Immunity, 2002. 70(2): p. 631-641.

**Table captions:**

**Table 1: List of primers used for the detection of the enterotoxins, hemolysin, fibronectin-binding protein, and exfoliative toxins genes.**

**Table 2: Analysis of the different virulence-associated genes expressed by *S. haemolyticus****.*

Table 3: Summary of the frequency of virulence genes and invasion efficiencies of the *S. haemolyticus* strains.

**Table 1: List of primers used for the detection of the enterotoxins, hemolysin, fibronectin-binding protein, and exfoliative toxins genes.**

| ***Group name*** | ***Primer name*** | ***Gene name*** | ***Sequence*** | ***Size (bp)*** | ***References*** |
| --- | --- | --- | --- | --- | --- |
| *Enterotoxins* | *sea-1*  *sea-2* | *Enterotoxin A* | TTGGAAACGGTTAAAACGAA  GAACCTTCCCATCAAAAACA | 120 | [[60](#_ENREF_60)] |
|  | *seb-1*  *seb-2* | *Enterotoxin B* | TCGCATCAAACTGACAAACG  GCAGGTACTCTATAAGTGCC | 478 |  |
|  | *sec-1*  *sec-2* | *Enterotoxin C* | GACATAAAAGCTAGGAATTT  AAATCGGATTAACATTATCC | 257 |  |
|  | *sed-1*  *sed-2* | *Enterotoxin D* | CTAGTTTGGTAATATCTCCT  TAATGCTATATCTTATAGGG | 317 |  |
|  | *see-1*  *see-2* | *Enterotoxin E* | CAAAGAAATGCTTTAAGCAATCTTAGGCCAC  CTTACCGCCAAAGCTG | 482 |  |
|  | *seg-1*  *seg-2* | *Enterotoxin G* | AATTATGTGAATGCTCAACCCGATC  AAACTTATATGGAACAAAAGGTACTAGTTC | 642 |  |
|  | *seh-1*  *seh-2* | *Enterotoxin H* | CAATCACATCATATGCGAAAGCAG  CATCTACCCAAACATTAGCACC | 376 |  |
| *Hemolysin* | *hla_haem-1*  *hla_haem-2* | *α-hemolysin* | TGGGCCATAAACTTCAATCGC  ACGCCACCTACATGCAGATTT | 72 |  |
| *Fibronectin-binding protein* | *fnbA* | *Fibronectin-Binding Proteins A* | CCCTCTTCGTTATTCAGCC  CAGGAGGCAAGTCACCTTG | 422 | [[73](#_ENREF_73)] |
|  | *fnbB* | *Fibronectin-Binding Proteins B* | TAAATCAGAGCCGCCAGTGGAG  GTCCTTGCGCTTGACCATGTTC | 416 | [[74](#_ENREF_74)] |
| *Exfoliative toxins* | *eta F*  *eta R* | exfoliative toxins A | ACTGTAGGAGCTAGTGCATTTGT  TGGATACTTTTGTCTATCTTTTTCATCAAC | 190 | [[75](#_ENREF_75)] |
|  | *etb F*  *etb R* | exfoliative toxins B | CAGATAAAGAGCTTTATACACACATTAC  AGTGAACTTATCTTTCTATTGAAAAACACTC | 612 |  |

**Table 2: Analysis of the different virulence-associated genes expressed by *S. haemolyticus****.*

| **Strain** | ***Enterotoxins*** | | | | | | | ***Hemolysin*** | ***Exfoliative toxins*** | | ***Fibronectin-binding protein*** | | **Adhesion to**  **PHSF cells*** |
| --- | --- | --- | --- | --- | --- | --- | --- | --- | --- | --- | --- | --- | --- |
|  | *sea* | *seb* | *sec* | *sed* | *see* | *seg* | *seh* | *hla* | *eta* | *etb* | *fnbA* | *fnbB* |  |
| SH 1 | - | - | + | - | - | - | + | + | - | - | - | - | + |
| SH 2 | - | - | + | - | - | + | + | + | - | - | + | + | ++++ |
| SH 3 | - | - | - | - | - | + | + | + | - | - | - | + | ++ |
| SH 4 | - | - | - | - | - | - | - | + | - | - | - | + | ++++ |
| SH 5 | + | - | - | - | - | - | - | - | - | - | - | + | ++ |
| SH 6 | + | - | - | - | - | - | - | - | - | - | - | + | ++++ |
| SH 7 | + | - | - | - | - | + | - | + | - | - | + | + | ++ |
| SH 8 | - | - | - | - | - | - | - | + | - | - | - | + | +++ |
| SH 9 | - | - | + | - | - | + | - | + | - | - | + | + | ++++ |
| SH 10 | + | - | + | + | - | + | - | + | - | - | + | + | ++ |

* Adherent *S. haemolyticus* were counted microscopically (magnification x100) in 20 random microscopic fields and averaged. Very strong adhesion (++++) represented > 2500 adherent bacterial cells; strong adhesion (+++) represented 2500-1000 adherent bacteria; moderate adhesion (++) represented 1000-100 adherent bacteria and weak adhesion (+) represented less than 100 adherent bacteria.

Table 3: Summary of the frequency of virulence genes and invasion efficiencies of the *S. haemolyticus* strains

| **Bacterial strain** | **Number of virulence genes** | **Frequency of FITC^+^ PHSF cells** | **Percentage of internalized bacteria relative to added cells±SD** |
| --- | --- | --- | --- |
| SH 1 | 3 | 0.91 | 0.1±0.2 |
| SH 2 | 6 | 23.5 | 2±0.3 |
| SH 3 | 4 | 21.0 | 1.5±0.35 |
| SH 4 | 2 | 20.4 | 1.55±0.15 |
| SH 5 | 2 | 21.3 | 1.6±0.08 |
| SH 6 | 2 | 22.5 | 1.6±0.07 |
| SH 7 | 5 | 24.7 | 2.05±0.09 |
| SH 8 | 2 | 23.4 | 1.65±0.09 |
| SH 9 | 5 | 25.0 | 1.6±0.11 |
| SH10 | 7 | 26.9 | 2.5±0.07 |
| Control SH | ND* | 1.24 | 0.25±0.1 |

ND; Not determined


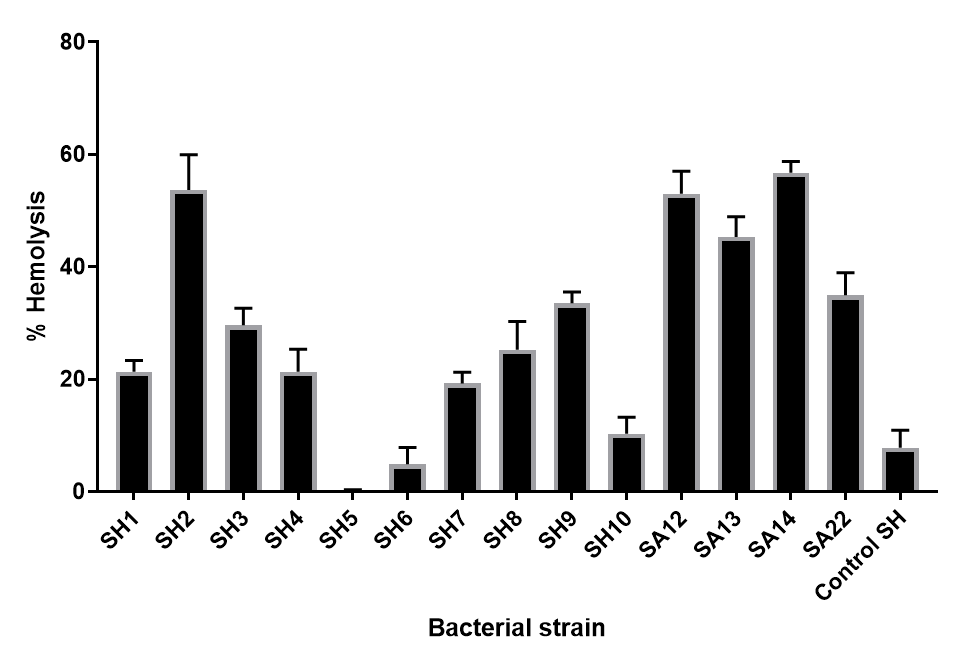


Figure 1: Hemolytic activities of clinical *S. haemolyticus* isolates compared to *S. aureus* and control *S. haemolyticus* (ATCC 29970) after 5 h of incubation. SH; *S. haemolyticus*, SA; *S. aureus*. Depicted are the mean of 3 independent experiments ± standard deviation.


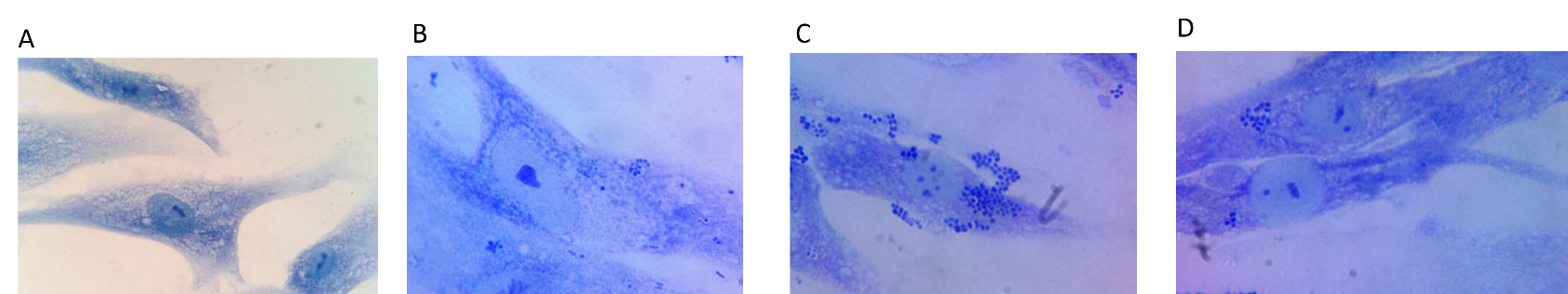


Figure 2: Bacterial adhesion to PHSF cells as detected by Giemsa stain. After 3 h in culture, PHSF cells were challenged with different *S. haemolyticus* isolates. (A) Non-infected fibroblast cells, (B) Fibroblasts infected with *S. haemolyticus* isolate with low adhesion capacity, (C) Fibroblasts infected with *S. haemolyticus* isolate with high adhesion capacity, (D) Fibroblasts infected with the control *S. haemolyticus* strain (ATCC 29970) showing low adhesion capacity.


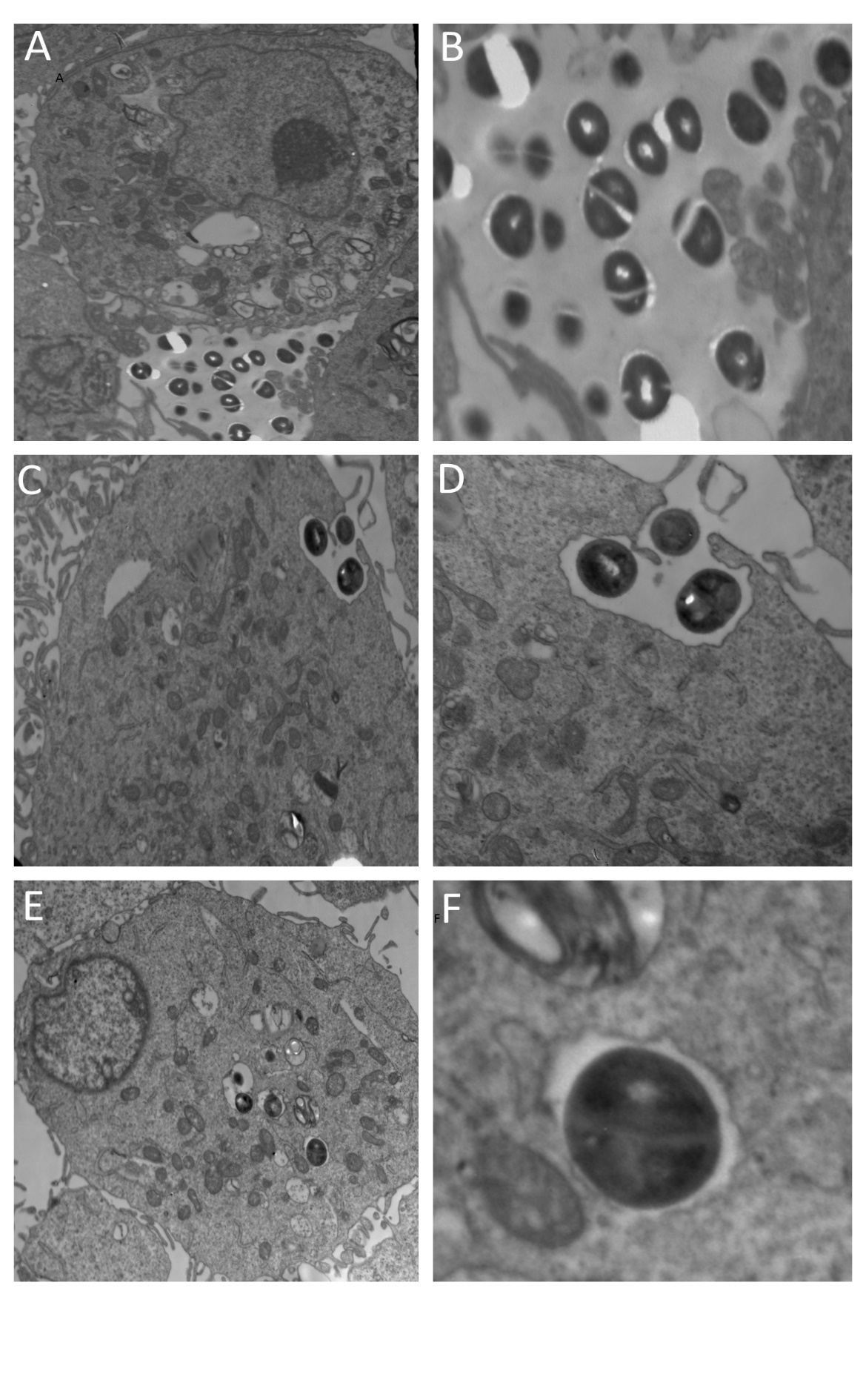


Figure 3: The PHSF cells were challenged with *S. haemolyticus* at an MOI of 10 and examined with TEM. (A) After 15 min of infection, extracellular bacteria were observed (3600 x) which were further magnified (14,000 x) in (B). (C) After 1 h of infection, bacteria invaded the cells and became engulfed (3600 x). (D) Initialization of phagocytosis. (E) After 90 min, *S. haemolyticus* entered the PHSF cells and become localized in vacuoles (3600 x). (F) Proliferating bacteria are shown inside the vacuole.

***
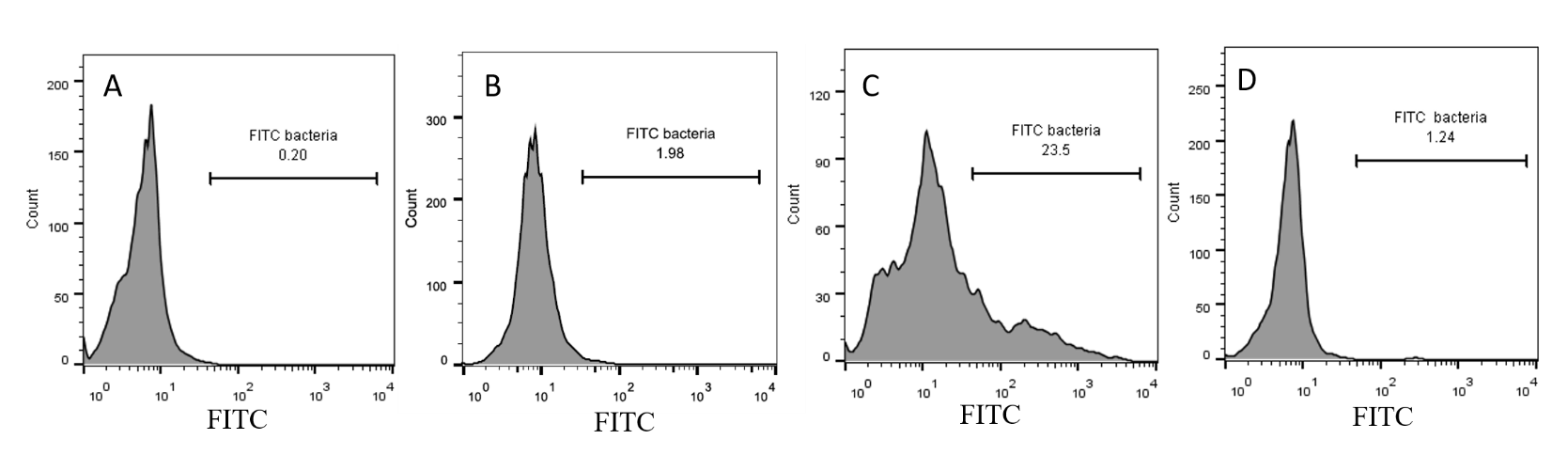
***

Figure 4: Invasion of PHSF cells by FITC-labelled bacteria. Comparative histograms represent results of flow cytometry analysis of (A) control non-infected cells (mock), (B) poorly invasive strain of *S. haemolyticus* (SH1), (C) highly invasive strain of *S. haemolyticus* (SH10), (D) Control *S. haemolyticus* strain (ATCC 29970). The figure shows a representative of three independent experiments.


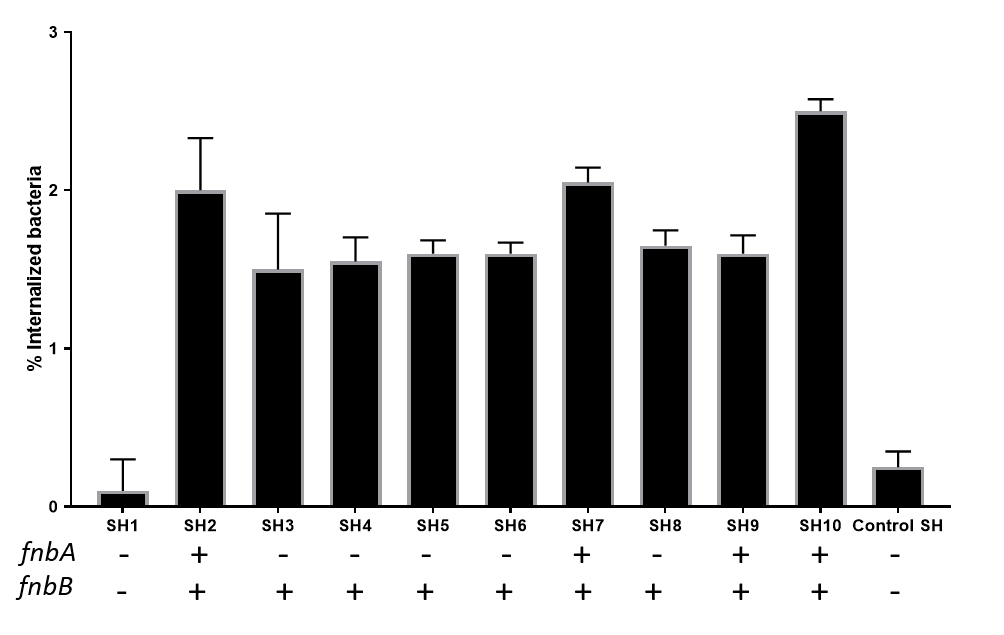


Figure 5: Percentage of internalized *S. haemolyticus* in the PHSF cells and the distribution of the *fnBP* genes.

**Figure 6:** **Effect of *Staphylococci* on the proliferation rate of PHSFcells measured by MTT assay**. Data are represented as the mean of 3 experiments ± standard error.


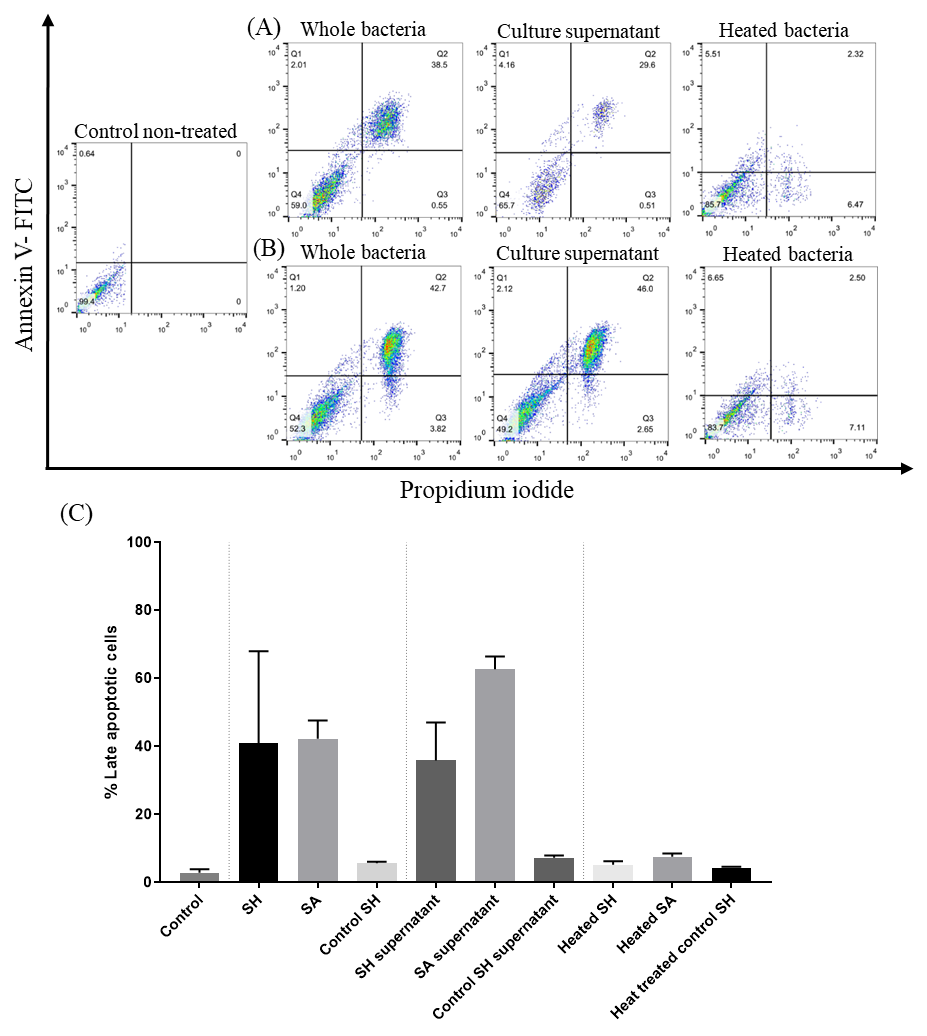


Figure 7: Flow cytometry analysis showing the cytotoxic effect of SH and SA on the PHSF cells. Cells were treated with indicated microbial products and stained after 24 h of incubation with Annexin V and PI. (A) Cells were infected with SH (upper panel), while in (B) cells were infected with SA (lower panel). (C) The mean of the cytotoxic activities of different tested strains on the PHSF cells ± SD. SH, *S. haemolyticus*; SA, *S. aureus*.

*
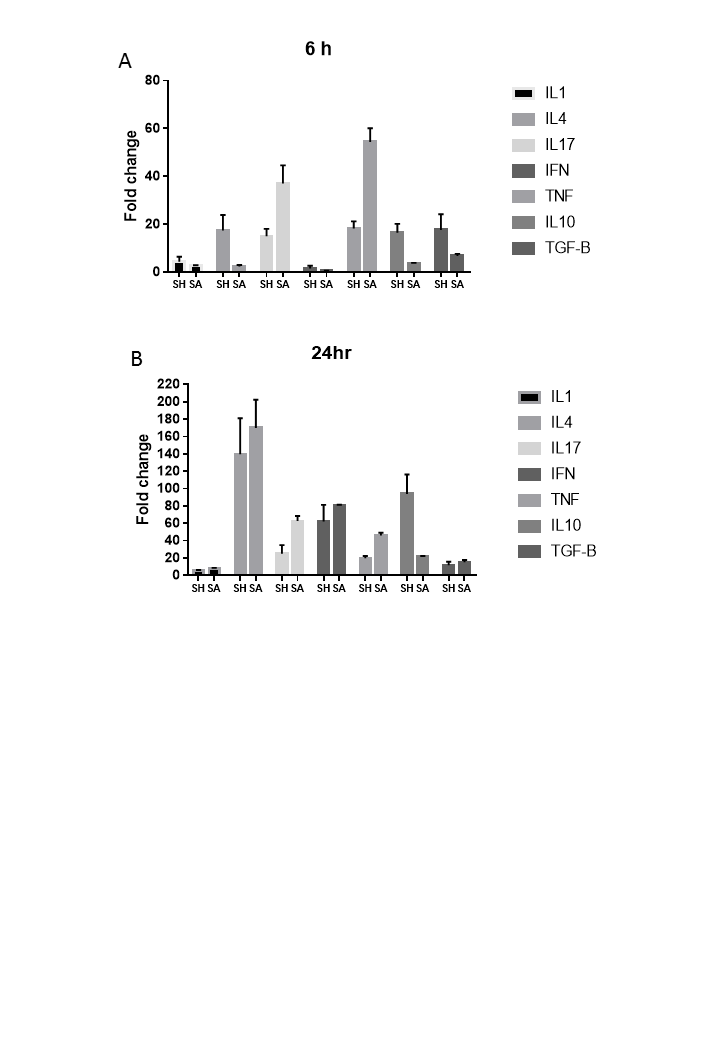
*

**Figure 8:** **Cytokine expression profile of PBMCs incubated with *S. haemolyticus* and *S. aureus* for 6 and 24 h**. The figure shows the mean of 3 independent experiments ± standard error.

Figure 1: Hemolytic activities of clinical *S. haemolyticus* isolates compared to *S. aureus* and control *S. haemolyticus* (ATCC 29970) after 5 h of incubation. SH; *S. haemolyticus*, SA; *S. aureus*. Depicted are the mean of 3 independent experiments ± standard deviation.

Figure 2: Bacterial adhesion to PHSF cells as detected by Giemsa stain. After 3 h in culture, PHSF cells were challenged with different *S. haemolyticus* isolates. (A) Non-infected fibroblast cells, (B) Fibroblasts infected with *S. haemolyticus* isolate with low adhesion capacity, (C) Fibroblasts infected with *S. haemolyticus* isolate with high adhesion capacity, (D) Fibroblasts infected with the control *S. haemolyticus* strain (ATCC 29970) showing low adhesion capacity.

Figure 3: The PHSF cells were challenged with *S. haemolyticus* at an MOI of 10 and examined with TEM. (A) After 15 min of infection, extracellular bacteria were observed (3600 x) which were further magnified (14,000 x) in (B). (C) After 1 h of infection, bacteria invaded the cells and became engulfed (3600 x). (D) Initialization of phagocytosis. (E) After 90 min, *S. haemolyticus* entered the PHSF cells and become localized in vacuoles (3600 x). (F) Proliferating bacteria are shown inside the vacuole.

Figure 4: Invasion of PHSF cells by FITC-labelled bacteria. Comparative histograms represent results of flow cytometry analysis of (A) control non-infected cells (mock), (B) poorly invasive strain of *S. haemolyticus* (SH1), (C) highly invasive strain of *S. haemolyticus* (SH10), (D) Control *S. haemolyticus* strain (ATCC 29970). The figure shows a representative of three independent experiments.

Figure 5: Percentage of internalized *S. haemolyticus* in the PHSF cells and the distribution of the *fnBP* genes.

**Figure 6:** **Effect of *Staphylococci* on the proliferation rate of PHSFcells measured by MTT assay**. Data are represented as the mean of 3 experiments ± standard error.

Figure 7: Flow cytometry analysis showing the cytotoxic effect of SH and SA on the PHSF cells. Cells were treated with indicated microbial products and stained after 24 h of incubation with Annexin V and PI. (A) Cells were infected with SH (upper panel), while in (B) cells were infected with SA (lower panel). (C) The mean of the cytotoxic activities of different tested strains on the PHSF cells ± SD. SH, *S. haemolyticus*; SA, *S. aureus*.

**Figure 8:** **Cytokine expression profile of PBMCs incubated with *S. haemolyticus* and *S. aureus* for 6 and 24 h**. The figure shows the mean of 3 independent experiments ± standard error.
